# A Phosphonate Natural Product Made by Pantoea ananatis is Necessary and Sufficient for the Hallmark Lesions of Onion Center Rot

**DOI:** 10.1128/mBio.03402-20

**Published:** 2021-02-02

**Authors:** Alexander L. A. Polidore, Lucia Furiassi, Paul J. Hergenrother, William W. Metcalf

**Affiliations:** a Department of Microbiology, University of Illinois at Urbana-Champaign, Urbana, Illinois, USA; b Department of Chemistry, University of Illinois at Urbana-Champaign, Urbana, Illinois, USA; c Carl R. Woese Institute for Genomic Biology, University of Illinois at Urbana-Champaign, Urbana, Illinois, USA; Mass General Hospital

**Keywords:** biosynthesis, *Pantoea*, natural products, onion, herbicide, phosphonate

## Abstract

Pantoea ananatis is the primary cause of onion center rot. Genetic data suggest that a phosphonic acid natural product is required for pathogenesis; however, the nature of the molecule is unknown. Here, we show that *P. ananatis* produces at least three phosphonates, two of which were purified and structurally characterized. The first, designated pantaphos, was shown to be 2-(hydroxy[phosphono]methyl)maleate; the second, a probable biosynthetic precursor, was shown to be 2-(phosphonomethyl)maleate. Purified pantaphos is both necessary and sufficient for the hallmark lesions of onion center rot. Moreover, when tested against mustard seedlings, the phytotoxic activity of pantaphos was comparable to the widely used herbicides glyphosate and phosphinothricin. Pantaphos was also active against a variety of human cell lines but was significantly more toxic to glioblastoma cells. Pantaphos showed little activity when tested against a variety of bacteria and fungi.

## Abstract (precis)

Pantoea ananatis is a significant plant pathogen that targets a number of important crops, a problem that is compounded by the absence of effective treatments to prevent its spread. Our identification of pantaphos as the key virulence factor in onion center rot suggests a variety of approaches that could be employed to address this significant plant disease.

## INTRODUCTION

*Pantoea* species have been recognized as plant pathogens since 1928 (1). These Gram-negative *Enterobacteriaceae* were originally classified as members of the genus *Erwinia* but were subsequently moved to *Pantoea* based on DNA hybridization experiments (2). Although many *Pantoea* species are benign or beneficial plant mutualists, strains of *P. ananatis* are consistently associated with harmful crop and forest infestations (3–7). Since 1983, the known hosts of *P. ananatis* have increased to 8 plant species in 11 countries, including important crops such as rice, corn, onions, melon, and pineapple (8). Upon plant infection, these bacteria cause internal rotting, dieback, and blight, resulting in severe economic losses. In addition to primary infection in the field, significant postharvest losses, as observed in onion center rot, have also been reported (9). Moreover, this plant pathogen can also infect humans and insects, which serve as vectors for plant infection (10–12). Thus, there is a compelling need to understand *P. ananatis* pathogenesis to help address its epidemic spread among essential food crops.

Despite the economic and food safety implications of *P. ananatis* infection, the mechanisms of plant pathogenicity have only recently been investigated. Comparative genomic analyses revealed substantial diversity between *P. ananatis* strains, which may account for their ability to colonize and thrive in so many different hosts (13, 14). The pathogenicity determinants encoded by diverse *P. ananatis* genomes include quorum sensing systems, type VI secretion systems, motility factors, cell wall-degrading enzymes, and thiosulfinate resistance alleles (15–17). In 2019, a novel pathogenicity determinant for onion center rot was revealed by comparison of the genomic sequences of two pathogenic and two nonpathogenic *P. ananatis* strains (18). This approach identified a genomic island designated “HiVir,” which was subsequently shown to be present in 14 pathogenic strains and absent in 16 nonpathogenic strains using a PCR-based screen. The HiVir locus encodes an 11-gene operon (here, designated *hvr*) that was suggested to encode a biosynthetic pathway for an unknown phosphonic acid natural product based on the presence of a putative *pepM* gene. This gene encodes the enzyme phosphoenolpyruvate (PEP) phosphonomutase, which catalyzes the first step in all characterized phosphonate biosynthetic pathways and which has been extensively used as a genetic marker for the ability to produce phosphonic acid metabolites (19, 20). Deletion of *pepM* in *P. ananatis* OC5a resulted in a strain with severely attenuated pathogenicity in Allium cepa (onion), demonstrating a required role for the *hvr* operon in onion center rot. Based on this finding, Asselin et al. (18) suggested that a small molecule phosphonate is involved in plant disease caused by *P. ananatis*.

Phosphonates, defined by the presence of chemically stable carbon-phosphorus bonds, are an underdeveloped class of bioactive molecules with significant applications in both medicine and agriculture. The bioactivity of these molecules results from their structural similarity to phosphate esters and carboxylic acids, which allows them to bind enzymes that act on analogous substrates, thus inhibiting enzyme activity. A prominent example is the manmade herbicide glyphosate, which was first synthesized by chemists in the 1950s. The phytotoxicity of glyphosate is due to its inhibition of 5-enolpyruvylshikimate-3-phosphate (EPSP) synthase, a key enzyme in the biosynthesis of aromatic amino acids in plants (21). Significantly, enzyme inhibition by individual phosphonates is quite specific and typically confined to enzymes that act on chemically homologous substrates. Accordingly, a phosphonate can be toxic to one group of organisms while remaining innocuous to another. Thus, depending on the organism in which the target enzyme is found, phosphonates find applications as specific antibacterial, antifungal, antiparasital, and herbicidal compounds (22–24). Given the ubiquitous occurrence of phosphate esters and carboxylic acids in metabolism, the range of potential biological targets for phosphonate inhibitors is vast. Indeed, as demonstrated by the number of organisms known to produce bioactive phosphonates, nature has often capitalized on this metabolic Achilles’ heel. Examples include phosphinothricin tripeptide and fosmidomycin, produced by members of the genus *Streptomyces*, which have potent herbicidal and antimicrobial activity due to their inhibition of the essential enzymes glutamate synthase and deoxyxylulose-5-phosphate reductoisomerase, respectively (25–27). Nature also makes use of the fact that the C-P bond is highly stable and resistant to both chemical and enzymatic degradation. Accordingly, many organisms replace labile biomolecules such as phospholipids and phosphate ester-modified exopolysaccharides with analogous phosphonates (28, 29).

Considering their useful biological properties, it is not surprising that biosynthesis of phosphonate compounds is common among microbes. Based on the presence of *pepM* in sequenced genomes and metagenomes, ca. 5% of all bacteria possess the capacity for phosphonate biosynthesis (30). Biosynthetic gene clusters that include *pepM* are known to direct the biosynthesis of phosphonolipids, phosphonoglycans, and a wide variety of small-molecule secondary metabolites (22). Like the streptomycete-derived natural products described above, many of these small-molecule phosphonates are bioactive. Although considerable progress has been made in understanding the bioactivity and biosynthesis of small-molecule phosphonates, only a fraction of the observed *pepM*-encoding gene clusters have been characterized. Thus, the extent of phosphonate chemical diversity in nature has yet to be established.

Consistent with the idea that phosphonate biosynthesis is common in nature, it has also been observed that about 30% of sequenced bacterial genomes contain genes for phosphonate catabolism, which allows their use as sources of phosphorus, carbon or nitrogen (31–33). Genes encoding the carbon-phosphorus (C-P) lyase system, which catalyzes a multistep phosphonate degradation pathway with broad substrate specificity, are particularly common in bacteria (33). Other examples include the enzyme phosphonatase, which is specific for aminoethylphosphonate, and a recently characterized oxidative pathway for use of hydroxymethylphosphonate (34, 35).

Although the indirect evidence for the involvement of a bioactive phosphonate in *P. ananatis* pathogenesis is strong, this has yet to be established. Here, we show that the *hvr* operon indeed encodes enzymes responsible for production of a small-molecule phosphonate, which we show to be 2-[hydroxy(phosphono)methyl]maleate. The purified molecule, which we have designated pantaphos, has significant herbicidal activity and is able to produce the characteristic lesions of onion center rot in the absence of *P. ananatis*. Accordingly, this novel phosphonate natural product is both necessary and sufficient for onion rot pathogenesis. In addition, pathogenicity is enhanced in strains lacking phosphonate catabolism, suggesting that endogenous catabolism of pantaphos attenuates virulence.

## RESULTS

### Phosphonate metabolism in *P. ananatis* isolates.

Although previous studies correlating the *hvr* gene cluster and onion pathogenicity examined 30 *P. ananatis* strains via PCR, only a few these had fully sequenced genomes at the time of the study (18). To expand our understanding of phosphonate metabolism in this species, we searched the currently available set of 65 *P. ananatis* genomes, which includes 6 from the previous study, for the presence of genes associated with phosphonate biosynthesis and catabolism (Data Set S1). Based on the presence of the *pepM* gene, 33 (51%) of these strains have the genetic capacity to produce phosphonate metabolites, with 26 strains encoding a single *pepM* and 7 containing two copies. Four different phosphonate biosynthetic gene clusters were identified, with the *hvr* operon being the most common (28 strains). Eight strains carry a seven-gene phosphonate biosynthetic locus that is unrelated to characterized *pepM* gene clusters (Fig. S1, cluster A). Four of these strains also possess the *hvr* locus at a distal site on the chromosome. Three strains, all of which also carry the *hvr* locus, carry a gene cluster that resembles the phosphonoglycan biosynthetic locus in *Glyomyces* sp. strain NRRL B-16210 (Fig. S1, *pgb* gene cluster) (29). Lastly, a single strain, which lacks the *hvr* operon, carries a novel nine-gene *pepM-*encoding cluster (Fig. S1, cluster B). In addition to the putative phosphonate biosynthetic loci, all strains carry a copy of the *phn* operon, which encodes the carbon-phosphorus lyase system used for phosphonate catabolism (Fig. S1, *phn* gene cluster) (36). Genes encoding alternative phosphonate catabolic pathways were not observed.

10.1128/mBio.03402-20.4 FIG S1 Phosphonate-related gene clusters in *P. ananatis*. Four distinct phosphonate metabolism gene clusters identified in a search of 65 *P. ananatis* genomes are shown. The putative functions of each gene were assigned based on homology identified in BLAST searches to genes of known function. The presence/absence of each cluster in each genome is detailed in Data Set S1. Download FIG S1, EPS file, 1.5 MB. Copyright © 2021 Polidore et al. 2021 Polidore et al. This content is distributed under the terms of the Creative Commons Attribution 4.0 International license.

10.1128/mBio.03402-20.1 DATA SET S1 Phosphonate-related gene clusters associated with various pathogenic and nonpathogenic *P. ananatis* strains. Download Data Set S1, XLSX file, 0.02 MB. Copyright © 2021 Polidore et al. 2021 Polidore et al. This content is distributed under the terms of the Creative Commons Attribution 4.0 International license.

### Role of phosphonate metabolism in onion center rot.

**FIG 1:**
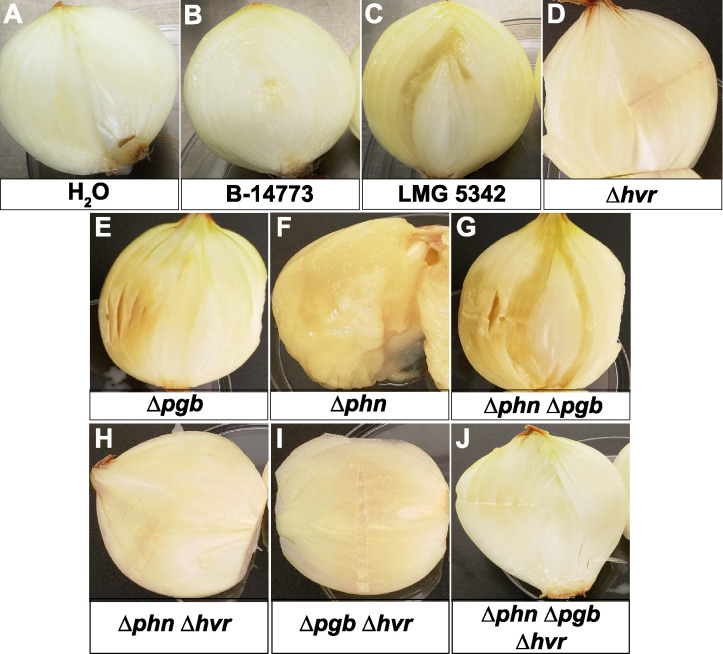
Onion center rot phenotype of *P. ananatis* strains used in the study. Surface-sterilized onion bulbs were inoculated as indicated in each panel and then incubated at 30°C for 14 days prior to sectioning to reveal the center rot phenotype. (A to C) Inoculations of a sterile water control and wild-type strains. (D to J) Inoculations with mutant derivatives of *P. ananatis* LMG 5342.

To examine the broader role of phosphonate metabolism in onion pathogenesis, we characterized *P. ananatis* LMG 5342, which carries the *hvr*, *pgb*, and *phn* loci, using *P. ananatis* B-14773, which does not encode any phosphonate biosynthetic genes, as a control. Because the pathogenicity of these strains has not been established, initial experiments were conducted to assess their ability to cause onion rot. Consistent with the previously observed correlation between the presence of the *hvr* locus and pathogenesis, we observed onion center rot in bulbs inoculated with strain LMG 5342, but not in bulbs inoculated with B-14773 (Fig. 1).

To verify that the observed pathogenic phenotype required *hvr*, and to examine whether the additional phosphonate metabolism genes play a role in pathogenesis, we constructed a series of LMG 5342 mutants lacking the *hvr*, *pgb*, and *phn* loci in all possible combinations and scored their ability to cause onion rot (Fig. 1). The center rot phenotype was observed in all mutants that retained the *hvr* locus and absent in all strains with the Δ*hvr* mutation. Therefore, as observed in *P. ananatis* OC5a (18), the *hvr* locus is necessary for onion pathogenicity in strain LMG 5342. In contrast, the *pgb* locus does not significantly contribute to pathogenicity, because the onion rot phenotype for mutants lacking these genes was identical to each of the otherwise isogenic strains. Interestingly, strains with intact *hvr* and *phn* loci were attenuated relative to those with the Δ*phn* mutation, suggesting that endogenous phosphonate catabolism minimizes virulence (Fig. 1).

### Production of phosphonic acids in *P. ananatis* LMG 5342.

To examine whether phosphonates are actually produced by *P. ananatis* LMG 5342, we grew the wild-type strains in a variety of liquid and solid media. Spent media were then concentrated and screened for the presence of phosphonates using ^31^P nuclear magnetic resonance (NMR), which allows relatively sensitive detection of molecules containing a carbon-phosphorus (C-P) bond, even in complex mixtures containing phosphate and phosphate esters (37). In no case did we observe signals consistent with the presence of phosphonates. We suspected that our inability to detect phosphonates in spent media was due to poor expression of the *hvr* operon in the media we employed. Thus, we conducted similar experiments in media supplemented with onion extract, with the idea that a plant metabolite was required to induce expression of the *hvr* locus; however, we failed to detect phosphonates in these media as well.

**FIG 2:**
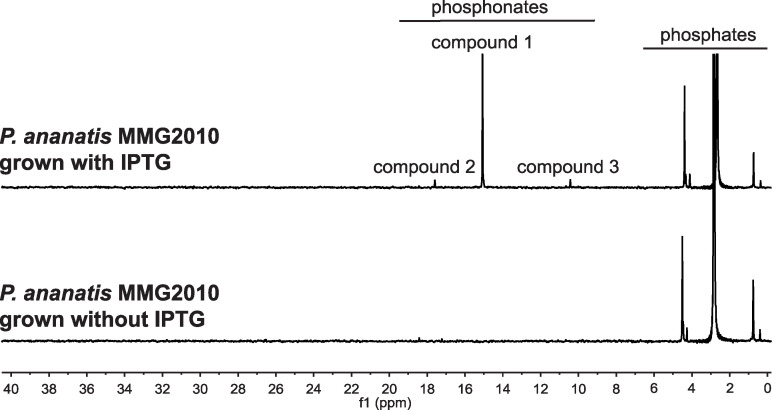
Production of phosphonates is correlated with expression of the *hvr* operon. *P. ananatis* MMG2010, which expresses the *hvr* operon from an IPTG-inducible promoter (see Fig. S2), was grown in glycerol minimal medium with and without IPTG. Spent media were then concentrated and analyzed by ^31^P-NMR. Phosphonic acids typically produce peaks in the 5 to 30 ppm range, whereas peaks from phosphate and its esters and anhydrides typically have chemical shifts in the <5 ppm range.

To circumvent issues arising from native gene regulation, we constructed a strain that expresses the *hvr* operon from a strong, isopropyl-β-d-1-thiogalactopyranoside (IPTG)-inducible promoter (Fig. S2). To avoid complications caused by the other phosphonate metabolic genes, this strain also carried the Δ*phn* and Δ*pgb* mutations. After growth of this recombinant strain in media with IPTG, three distinct ^31^P NMR peaks were observed (Fig. 2). The chemical shifts of these peaks (*δ* 17.6, *δ* 15.0, and *δ* 10.4 ppm) are consistent with the presence of molecules containing C-P bonds. These peaks were not observed after growth in media without IPTG. Thus, putative phosphonates are produced only when the *hvr* operon is expressed. After optimization of the medium and growth conditions, the IPTG-inducible strain catalyzed nearly complete conversion of phosphate to biomass and phosphonates, with final concentrations of approximately 0.653, 0.080, and 0.039 mM for compounds 1 (*δ* 17.6), 2 (*δ* 15.0), and 3 (*δ* 10.4 ppm), respectively (Fig. S3).

10.1128/mBio.03402-20.5 FIG S2 Construction of a *P. ananatis* LMG5342 derivative with IPTG-inducible *hvr* expression. Plasmid pAP01, which carries the *hvrA* gene under control of the IPTG-inducible Ptac promoter, was transferred to *P. ananatis* MMG1998 via conjugation from an E. coli donor. Because pAP01 is incapable of autonomous replication in *P. ananatis*, kanamycin-resistant (conferred by the *aph* gene) excongugants can only be obtained by chromosomal integration of the plasmid via homologous recombination (depicted as dotted lines). The resulting strain, *P. ananatis* MMG2010, expresses the entire *hvr* operon from the Ptac promoter. The positions of the native *hvr* promoters are shown as unlabeled bent arrows. Download FIG S2, EPS file, 1.8 MB. Copyright © 2021 Polidore et al. 2021 Polidore et al. This content is distributed under the terms of the Creative Commons Attribution 4.0 International license.

10.1128/mBio.03402-20.6 FIG S3 Quantitative ^31^P-NMR after growth of *P. ananatis* MMG2010 in an optimized medium. Spent media were concentrated 5-fold and analyzed by quantitative ^31^P-NMR after addition of 0.5 mM dimethylphosphinate as an internal standard. The relative abundances used for quantification are shown in blue. The structures of each compound (to the extent known) are indicated. Download FIG S3, EPS file, 0.9 MB. Copyright © 2021 Polidore et al. 2021 Polidore et al. This content is distributed under the terms of the Creative Commons Attribution 4.0 International license.

### Structure elucidation of *hvr*-related phosphonates.

**FIG 3:**
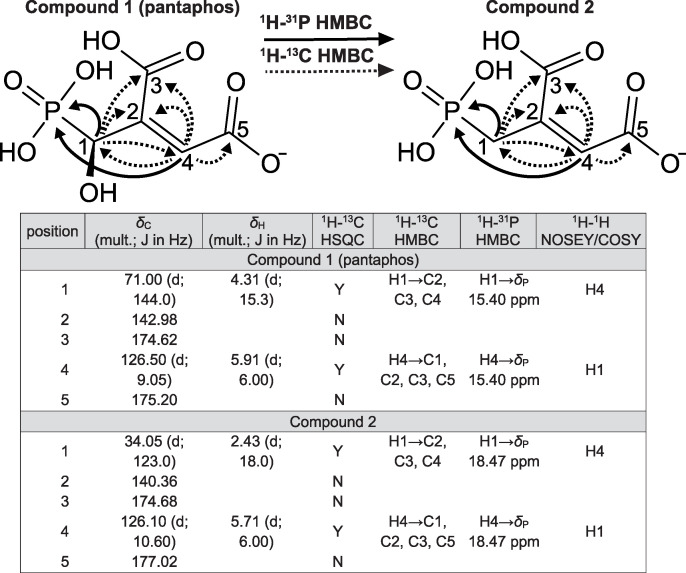
Summary of NMR data supporting the structure of compounds 1 and 2. Primary data and full description of structural elucidation is provided in the Data Set S1.

From a 3.2-liter culture grown using these optimized conditions, we were able to isolate 8.9 mg of pure compound 1 and a small amount (<600 μg) of pure compound 2. Compound 3 (*δ_P_* 10.42 ppm) was not obtained, because it is unstable at the low pH used during affinity chromatography (data not shown). The structures of compounds 1 and 2 were elucidated using a series of one- and two-dimensional carbon, phosphorus, and proton NMR experiments (summarized in Fig. 3; the full data set is described in Data Set S2). Compound 1, which we designated pantaphos, was shown to be 2-[hydroxy(phosphono)methyl]maleate. Compound 2 (2-phosphonomethylmaleate) has a nearly identical structure but lacks the hydroxyl-group, suggesting that it may be an intermediate in the biosynthesis of pantaphos (Fig. 3). High-resolution mass spectrometry data of the purified compounds are fully consistent with these structures (Data Set S2).

### Lesions of onion center rot are caused by pantaphos.

**FIG 4:**
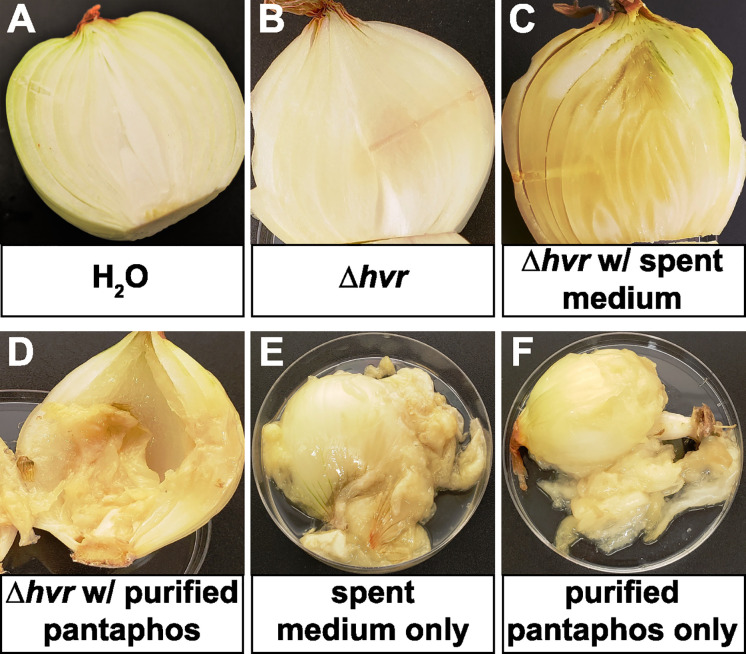
Chemical complementation of the Δ*hvr* onion rot phenotype by *P. ananatis* phosphonates. Surface-sterilized onion bulbs were inoculated as indicated in each panel and then incubated at 30°C for 14 days prior to sectioning to reveal the center rot phenotype. (A and B) Inoculations of a sterile water control and the Δ*hvr* mutant. (C and D) Inoculations with the *hvr* mutant supplemented with spent medium from an IPTG-induced culture of the phosphonate-producing strain *P. ananatis* MMG2010 or purified pantaphos. (E and F) Inoculations of onion with sterile spent medium or purified pantaphos in the absence of bacteria.

To investigate the role of *hvr*-associated phosphonates in pathogenesis, we repeated the onion rot assay using concentrated culture supernatants or purified pantaphos in the presence and absence the *P. ananatis* Δ*hvr* mutant (Fig. 4). (Pure compound 2 was not obtained in sufficient quantities to allow bioactivity testing.) As described above, onions inoculated with *P. ananatis* Δ*hvr* mutants showed minimal damage. However, when concentrated spent medium from IPTG-induced cultures of the phosphonate overproducing strain was coinoculated with the Δ*hvr* mutant, center rot was again observed. Similarly, coinoculation with purified pantaphos also resulted in onion rot. Significantly, onions injected with either concentrated spent medium or purified pantaphos showed severe onion rot lesions in the absence of bacteria. The occurrence of center rot was dose-dependent, with the characteristic lesions observed using as little as 90 μg (0.40 μmol) of pantaphos (Fig. S4). Therefore, pantaphos is both necessary and sufficient to cause the center rot lesions in onions.

10.1128/mBio.03402-20.7 FIG S4 Dose dependency of onion center rot lesions with purified pantaphos. Surface-sterilized onion bulbs were injected with pantaphos concentration as indicated in each panel and then incubated at 30°C for 14 days prior to sectioning to reveal the center rot phenotype. Download FIG S4, EPS file, 2.1 MB. Copyright © 2021 Polidore et al. 2021 Polidore et al. This content is distributed under the terms of the Creative Commons Attribution 4.0 International license.

### Phytotoxic effects of pantaphos treatment are comparable to known herbicides.

**FIG 5:**
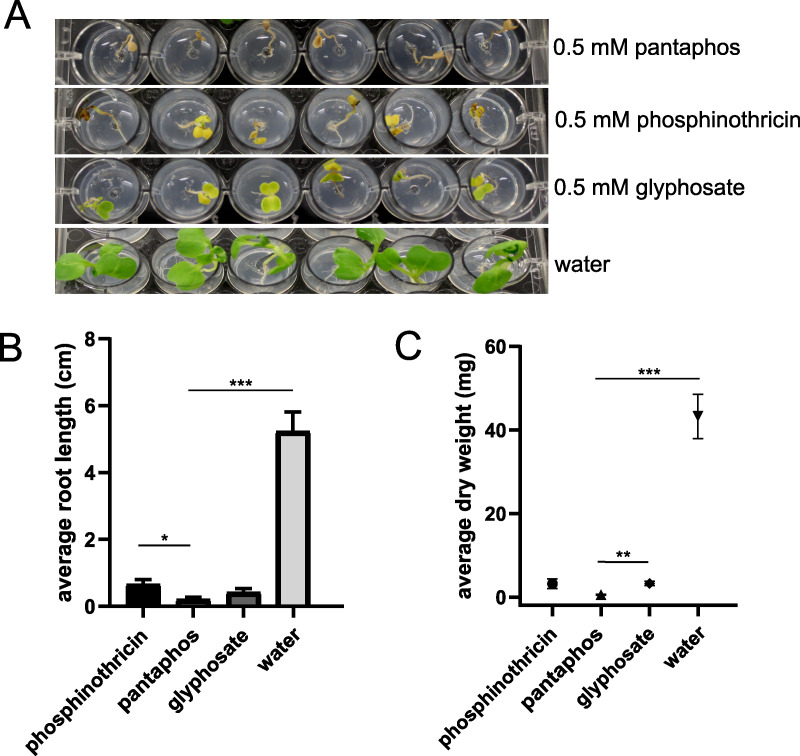
Phytotoxicity of pantaphos compared to known herbicides. Mustard seedlings were treated as indicated and incubated at 23°C under a 16-h light cycle for 7 days. (A) The observed phenotype after incubation with the indicated compounds. (B and C) Root length and dry weight of each replicate after incubation. Welch’s *t* test was performed to establish significant differences between the means of each treatment (***, *P* < 0.001; **, *P* < 0.01; *, *P* < 0.05; *n* = 6 per treatment). Error bars represent the standard error of the mean.

To test whether the phytotoxicity observed in onion bulbs could be extended to growing plants, we treated newly germinated mustard seedlings (*Brassica* sp.) with purified pantaphos using two well-characterized phosphonate herbicides (glyphosate and phosphinothricin) as controls (Fig. 5). After 7 days of growth, all three compounds caused a substantial reduction in root length and total dry weight of the seedlings relative to water-treated controls, with pantaphos being significantly more potent than phosphinothricin in the root length assay than glyphosate in the dry weight assays. The phytotoxicity of pantaphos was dose-dependent with significant activity at concentrations of 1.95 μM and above in the root length assay and above 31.3 μM in the dry weight assay (Fig. S5).

10.1128/mBio.03402-20.8 FIG S5 Herbicidal dose dependency of pantaphos treatment in mustard seedlings. Mustard seedlings were germinated and transplanted into mannitol-soy flour (MS) agar and then spotted with pantaphos concentration as indicated and incubated at 23°C under a 16-h light cycle for 7 days. (A) The plant phenotypes after incubation. (B and C) Root length (B) and dry weight (C) were measured for each plant replicate, and Welch’s *t* test was performed to calculate statistics between the means of the different treatments (***, *P* < 0.001; **, *P* < 0.05; *n* = 6 per treatment). Error bars represent the standard error of the mean. ns, not significant. Download FIG S5, EPS file, 2.7 MB. Copyright © 2021 Polidore et al. 2021 Polidore et al. This content is distributed under the terms of the Creative Commons Attribution 4.0 International license.

### Cytotoxic, antibacterial, and antifungal bioactivities of pantaphos.

To examine whether the bioactivity of pantaphos was specific to plants, we also conducted a series of bioassays against human cell lines, bacteria, and fungi. Pantophos showed modest cytotoxicity to several human cell lines (Table 1). With the exception of one ovarian cancer cell line (ES-2), which was unaffected at the maximum dose, the 50% inhibitory concentration (IC_50_) levels were roughly similar, in the range of 6.0 to 37.0 μM for each of the cell lines tested. One glioma cell line (A-172) was especially sensitive to pantaphos (IC_50_ of 1.0 μM). In contrast, the molecule had no effect on the growth of fungi in rich or minimal media, including Candida albicans, Aspergillus fumigatus, and two strains of Saccharomyces cerevisiae (Table 1). Similarly, a variety of Gram-negative and Gram-positive bacteria, including all of the so-called ESKAPE pathogens, were insensitive to pantaphos in both minimal and rich media (Table 1). To examine whether the insensitivity of Escherichia coli was due to a lack of transport, we also tested bioactivity using the phosphonate-specific bioassay strain E. coli WM6242, which carries two copies of an IPTG-inducible, broad substrate-specificity phosphonate transporter (38). This strain was insensitive to pantaphos, with or without IPTG induction, suggesting that the lack of bioactivity in E. coli is not due to poor transport of the molecule.

**TABLE 1:** Bioactivity of pantaphos against human cells and microorganisms

Human cell line	IC_50_ (μM) (E_max_ [%])^*a*^		
HOS (human osteosarcoma)	36.98 ± 6.28 (58)		
ES-2 (human ovarian cancer)	>100		
HCT-116 (human colon cancer)	10.42 ± 2.00 (59)		
A-549 (human lung carcinoma)	14.73 ± 0.61 (66)		
HFF-1 (human fibroblast cells)	6.69 ± 0.29 (85)		
A-172 (human glioma cancer)	1.01 ± 0.06 (99)		

Fungi	MIC (μM) for:^*b*^	
	Rich medium	Minimal medium
Candida albicans SN250	>125	NT^*c*^
Aspergillus fumigatus 1163	>125	>125
Saccharomyces cerevisiae X2180-1A	>125	>125
Saccharomyces cerevisiae BY4741	>125	>125

Bacteria	MIC (μM) for:^*d*^	
	Rich medium	Minimal medium
Enterococcus faecalis ATCC 19433	>200	NT^*c*^	
Staphylococcus aureus ATCC 29213	>200	NT^*c*^	
Klebsiella pneumoniae ATCC 27736	>200	>200	
Acinetobacter baumannii ATCC 19606	>200	>200	
Pseudomonas aeruginosa PAO1	>200	>200	
Escherichia coli ATCC 25922	>200	>200	
Salmonella enterica LT2	>200	>200	
Escherichia coli WM6242 -IPTG	>200	>200	
Escherichia coli WM6242 +IPTG	>200	>200	

a IC_50_, 50% inhibitory concentration determined using the alamarBlue method. E_max_, percentage cell death.

b MIC determined based on CLSI guidelines. Bioactivity in rich medium was determined using RPMI 1640 medium. Bioactivity in minimal medium was determined using YMM medium.

c NT, not tested because the organism does not grow in minimal medium.

d MIC determined based on CLSI guidelines. Bioactivity in rich medium was determined using Mueller-Hinton 2 medium, and bioactivity in minimal medium was determined using glucose-MOPS minimal medium.

### Putative functions of the *hvr*-encoded proteins and proposed pantaphos biosynthetic pathway.

**FIG 6:**
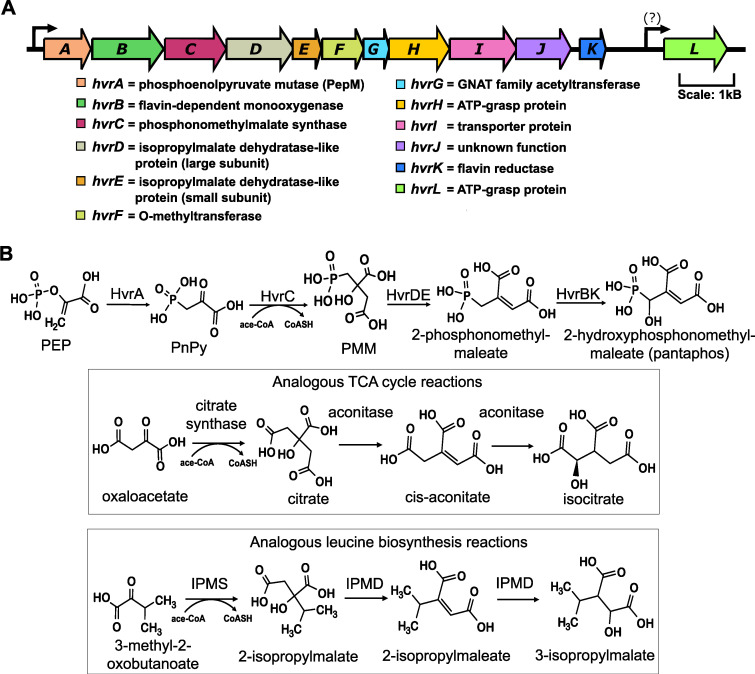
Hvr biosynthetic gene cluster in *P. ananatis* LMG 5342 and the proposed biosynthetic pathway. (A) The proposed protein functions of the Hvr BGC genes based on BLAST searches and conserved protein domain analyses. (B) The proposed biosynthetic pathway for pantaphos based on the structures of the *P. ananatis* phosphonates determined in this study and the biosynthetic logic of analogous reactions catalyzed by homologs of the Hvr proteins. PEP, phosphoenolpyruvate; PnPy, phosphonopyruvate; PMM, phosphonomethylmalate; IPMS, isopropylmalate synthase; IPMD, isopropylmalate dehydratase; ace-CoA, acetyl coenzyme A.

Combining the structures determined above with the proposed functions of the Hvr proteins suggests a reasonable biosynthetic route for the *P. ananatis* phosphonates (Fig. 6). As with most phosphonate natural products, the pathway begins with the rearrangement of phosphoenolpyruvate (PEP) to phosphonopyruvate (PnPy) catalyzed by the enzyme PEP mutase (39). In *P. ananatis*, this reaction would be catalyzed by the HvrA protein, which is highly homologous to known PEP mutases. Because the PEP mutase reaction is highly endergonic (ΔG ∼125 kJ/mol), subsequent steps must be highly favorable to drive net phosphonate synthesis (40). In the proposed pathway, this thermodynamic driving force is provided by the exergonic condensation of acetyl-CoA and phosphonopyruvate (PnPy) catalyzed by the HvrC protein, which is a homolog of the biochemically characterized phosphonomethylmalate (PMM) synthase involved in FR-900098 biosynthesis (41). PMM would then be dehydrated to form 2-phosphonomethylmaleate by HvrD and HvrE. These proteins are homologs of the small and large subunits of isopropylmalate dehydratase, respectively, which catalyze the isomerization of 3-isopropylmalate to 2-isopropylmalate via a dehydrated intermediate (2-isopropylmaleate) during leucine biosynthesis. We expect that HvrDE will not catalyze the full reaction but, rather, will stop at the dehydrated intermediate. Precedent for this partial reaction is found in the propionate catabolic pathway of some bacteria, which catalyze the dehydration of 2-methylcitrate to 2-methyl-*cis*-aconitate using a member of the isopropylmalate dehydratase family (42). Conversion of 2-phosphonomethylmaleate to pantaphos is likely to be catalyzed by HvrB, a homolog of the flavin-dependent monooxygenases, NtaA and ScmK (48% and 47% identity, respectively) (43, 44). Consistent with the idea that this is an oxygen-dependent reaction, we have observed that poorly aerated cultures accumulate 2-phosphonomethylmaleate instead of pantaphos (data not shown). Flavin-dependent monooxygenases often require a separate flavin reductase to provide the electrons needed for reduction of oxygen to water. We propose that this function is provided by HvrK, a member of the flavin reductase family that is 30% identical to NtaB, which serves this function in the analogous NtaA-catalyzed reaction (45). Finally, we suggest that the HvrI protein, which is a member of the major facilitator superfamily, is responsible for export of the phosphonate products (46, 47).

The proposed pathway for pantaphos biosynthesis uses only 7 of the 11 genes in the *hvr* operon (Fig. 6). Based on homology to proteins of known function, three of remaining genes are predicted to encode an *O*-methyltransferase (HvrF), an *N*-acetyltransferase (HvrG), and an ATP-Grasp family protein (HvrH). The final unassigned protein (HvrJ) has no characterized homologs, and thus, we cannot predict a function. A 12th protein that may, or may not, be part of the *hvr* operon also encodes an ATP-Grasp family protein (HvrL). Members of the ATP-Grasp family of enzymes often catalyze peptide bond formation (48). Accordingly, we suspect that peptidic derivatives of pantaphos may be produced by *P. ananatis*. Considering the absence of nitrogen in pantaphos, a peptidic derivative could also help explain the presence of the putative *N*-acetyltransferase HvrG, which might act as a self-resistance gene similar to the *pat* gene that confers self-resistance during biosynthesis of phosphinothricin tripeptide (49). Finally, the putative *O*-methyltransferase HvrF is highly homologous to *trans*-acontitate methyltransferase, which is thought to be involved in resistance to the spontaneously formed *trans* isomer of this tricarboxylic acid (TCA) cycle intermediate (50). An analogous role can be envisioned for HvrF if similar *trans* isomer side products are produced during pantaphos biosynthesis.

### Homologs of the *hvr* operon in other bacteria.

Asselin et al. noted the presence of gene clusters similar to the *hvr* operon in a number of bacterial genome sequences (18). We felt it was worth revisiting this analysis in the light of the structures identified above. A total of 33 related gene clusters were identified in the NCBI genomic sequence databases using a combination of bioinformatics approaches. Based on the arrangement and the presence or absence of orthologous genes, nine types of Hvr-like biosynthetic gene clusters were observed (Fig. S6) Putative orthologs of HvrA-F are conserved in all of these groups, with the exception of type VIII, which replaces the HvrDE proteins with a putative aconitase. As shown in Fig. 6, aconitase catalyzes a reaction that is essentially identical to the putative HvrDE reaction. Thus, we predict that the biosynthetic pathway encoded by the type VIII *hvr*-like gene cluster has identical intermediates produced by paralogous enzymes. A feature that differentiates the nine types of *hvr*-like clusters is the presence or absence of one or more ATP-Grasp proteins, suggesting that a variety of peptidic derivatives of pantaphos might be produced in nature. However, type IX, which lacks ATP-Grasp family proteins, likely has very different structural modifications compared to the other types, based on the presence of homologs to several additional enzyme families. Finally, the clusters also differ with respect to their putative export proteins and the presence/absence of a putative flavin reductase. The latter is not unexpected, as many flavin-dependent enzymes can utilize generic reductases encoded by unlinked genes (51, 52). Interestingly, the *hvr*-like clusters identified in our search were only found in a few lineages within the proteobacteria and actinobacteria (Fig. S7).

10.1128/mBio.03402-20.9 FIG S6 Homologous *hvr* biosynthetic gene clusters. Gene cluster homologs to the *P. ananatis* LMG 5342 *hvr* biosynthetic gene cluster were determined using a tblastn and MultiGeneBlast approach. The resulting homologous gene clusters were organized based on gene arrangement within the cluster and the presence of additional gene functions. Download FIG S6, EPS file, 1.0 MB. Copyright © 2021 Polidore et al. 2021 Polidore et al. This content is distributed under the terms of the Creative Commons Attribution 4.0 International license.

10.1128/mBio.03402-20.10 FIG S7 Distribution of homologous *hvr* biosynthetic gene clusters. A phylogenetic tree of strains with Hvr-like gene clusters was constructed using PATRIC Workspace. The *P. ananatis* strains from Data Set S1 are grouped together as indicated by the extended triangle node. Bootstrap values are shown where appropriate. IHA, insect host associated; PHA, plant host associated; SA, soil associated. Download FIG S7, EPS file, 2.0 MB. Copyright © 2021 Polidore et al. 2021 Polidore et al. This content is distributed under the terms of the Creative Commons Attribution 4.0 International license.

## DISCUSSION

The phosphonate natural products produced by *P. ananatis* LMG5342 are the principal virulence factor involved in onion center rot. Indeed, our data show that application of purified pantaphos produces identical lesions in the absence of bacteria. Although bioactive phosphonate natural products are well known, data supporting a direct role for these molecules in pathogenesis are rare. To date, the sole known example is a complex phosphonate-modified polysaccharide produced by Bacteroides fragilis, which was shown to promote abscess formation in the mammalian gut (28, 53). The demonstration that pantaphos is both necessary and sufficient for onion center rot adds a second phosphonate natural product to this short list and confirms the predicted function of the *P. ananatis hvr* locus proposed by Asselin et al. (18).

Despite the fact that unmodified pantaphos is phytotoxic, it seems likely that modified derivatives of the compound are also produced by *P. ananatis*. As described above, the *hvr* locus encodes two ATP-Grasp proteins. Members of this protein family often catalyze the ATP-dependent formation of peptide bonds, including those found in the peptidic phosphonate natural products rhizocticin, plumbemycin, and phosphonoalamide (54–56). Addition of amino acid substituents often enhances uptake of bioactive compounds. For example, phosphinothricin tripeptide is a potent antibacterial compound, while unmodified phosphinothricin has poor activity (57). It should be noted, however, that these transport-mediated effects are species specific. Thus, phosphinothricin and phosphinothricin tripeptide are equally effective herbicides (58). A particularly striking example of specificity conferred by amino acid substituents is seen in the bioactivity profile of rhizocticin and plumbemycin. These natural products have the same bioactive phosphonate warhead attached to different amino acids, which presumably governs their uptake by specific peptide transport systems. As a result, rhizocticin is a potent antifungal agent that lacks antibacterial activity, whereas plumbemycin is a potent antibacterial that lacks antifungal activity (59). Based on these precedents, we suspect that peptidic derivatives of pantaphos may be produced during plant infection that increase its potency or specificity for a particular target species, perhaps accounting for the unstable compound 3 we observed in spent media.

Interestingly, pantaphos is not the only phosphonate produced by *P. ananatis* species. At least four distinct phosphonate biosynthetic gene clusters can be found in the currently sequenced *P. ananatis* genomes. With the exception of the *hvr* operon, neither the structures nor the biological functions of the molecules produced by these gene clusters can be suggested at this time; however, our data clearly show that the *pgb* cluster is not required for onion center rot. Significantly, the strict correlation between plant pathogenicity and the presence of the *hvr* operon breaks down when larger numbers of *Pantoea* genomes are analyzed. Accordingly, *P. ananatis* strains PA4 and PNA 14-1 lack the *hvr* operon but still cause onion rot, whereas strains PANS 04-2, PNA 07–10, and PNA 200-7 carry the *hvr* locus but do not cause the disease. These data indicate that additional virulence traits are important for onion pathogenesis in *Pantoea* species. They also demonstrate the need for caution when using the presence/absence of the *hvr* cluster as a marker for plant pathogenicity.

Gene clusters similar to the *hvr* locus are relatively common in sequenced bacterial genomes; however, their phylogenetic distribution is rather restricted. Interestingly, many of the bacteria encoding these *hvr*-like gene clusters are associated with either plant or insect hosts. Homologs of the *hvr* operon are particularly abundant in members of the closely related *Photorhabdus* and *Xenorhabdus* genera. These bacteria have a unique lifestyle that relies on infection of an insect host via a nematode vector (60). Similarly, an *hvr* locus is found in Bartonella senegalensis OS02, a putative intracellular pathogen of mammals (61). Given the cytotoxic effects we observed in human cells, it is tempting to suggest that pantaphos-like compounds may be involved in bacterial pathogenesis in both insects and humans. Homologs of the *hvr* operon are also seen in several members of the actinobacteria, including species of *Streptomyces.* These bacteria, which are common epiphytes and endophytes, are known for their production of a wide array of secondary metabolites, including antibacterial, antifungal, and phytotoxic compounds (62, 63). Thus, it seems likely that bioactive pantaphos-like molecules are important for mutualistic interactions of actinobacteria as well.

The idea that pantaphos and its putative derivatives are involved in diverse mutualistic interactions begs the question of the biological target for these molecules. Among the organisms tested to date, only plants and human cells show significant sensitivity to pantaphos, while bacteria and fungi were completely insensitive to the molecule. A simple explanation for these observations is that the target is shared by plants and animals and absent from bacteria and fungi. Alternatively, the target could be shared by all organisms but be sufficiently different in bacteria and some fungi that it is not sensitive to the molecule. It is also possible that bacteria and some fungi share the ability to inactivate pantaphos or that they are incapable of transporting the molecule into their cells. Significant experimental effort, beyond the scope of this initial report, will be required to distinguish between these possibilities; however, at least for E. coli, the data suggest that a lack of transport is not responsible for the absence of bioactivity. Possible targets for pantaphos can be envisioned based on the structure of pantaphos, which is similar to a number of common metabolites, including citrate, isocitrate, aconitate, isopropylmalate, and maleate. Because most characterized phosphonates act as molecular mimics of normal cellular metabolites, this suggests the possibility that pantaphos may target the TCA cycle or leucine biosynthesis. Because maleic acid compounds are known to inhibit transaminases, it is also possible that the molecule inhibits the synthesis of another essential amine-bearing metabolite (64).

Finally, the studies described here have significant agricultural implications, which provide strong motivation for future studies on the biosynthesis and molecular target of pantaphos, as well as potential mechanisms for resistance to the compound. The identification of pantaphos as the principal virulence factor in onion rot suggests multiple approaches to deal with agricultural infestations of Pantoea ananatis. Because the molecule is required for virulence, it seems likely that inhibitors of the pantaphos biosynthetic pathway would prevent plant infection. It should also be possible to identify the molecular target of the compound in plants using purified or synthetic pantaphos, which would pave the way for development of crops with resistant alleles that would be immune to the disease. Plants expressing the putative pantaphos-modifying enzymes encoded by the *hvr* locus, or unrelated phosphonate catabolic genes, would also be expected to be specifically resistant to *Pantoea* infection. Finally, there is a desperate need to develop new treatments that are effective in combatting the alarming rise of herbicide-resistant weeds (65, 66). The potent phytotoxicity of pantaphos suggests that it may have agricultural utility similar to that of the widely used phosphonate herbicides glyphosate and phosphinothricin. The real possibility of developing pantaphos-resistant crops strengthens this idea, as does the absence of bioactivity toward bacteria and fungi, suggesting that the molecule would have minimal effects on the soil microbiome. A note of caution is appropriate, however, given the moderate cytotoxicity we observed in human cell lines.

## MATERIALS AND METHODS

### Bacteria, plasmids, and growth conditions.

The bacterial strains, plasmids, and primers used in this study are described in Table S1. General chemical and molecular biology reagents were purchased from Sigma-Aldrich or New England Biolabs. Phosphinothricin (glufosinate) was purchased from GoldBio (CAS no. 77182-82-2). Standard bacterial growth media were prepared as described (67–69). E. coli strains were typically grown at 37°C; *P. ananatis* strains were typically grown at 30°C. Phosphonate induction media (PIM) was prepared as follows: 8.37 g/liter MOPS (morpholinepropanesulfonic acid), 0.72 g/liter Tricine, 0.58 g/liter NaCl, 0.51 g/liter NH_4_Cl, 1.6 g/liter KOH, 0.1 g/liter MgCl_2_·6H_2_O, 0.05 g/liter K_2_SO_4_, and 0.2 g/liter K_2_HPO_4_ were dissolved in H_2_O and steam sterilized for 20 min at 121°C. After cooling, 1× sterile trace element solution was added [1,000× stock: 1.5 g/liter nitrilotriacetic acid trisodium salt, 0.8 g/liter Fe(NH_4_)_2 (_SO_4_)_2_·7H_2_O, 0.2 g/liter Na_2_SeO_3_, 0.1 g/liter CoCl_2_·6H_2_O, 0.1 g/liter MnSO_4_·H_2_O, 0.1 g/liter Na_2_MoO_4_·2H_2_O, 0.1 g/liter Na_2_WO_4_·2H_2_O, 0.1 g/liter ZnSO_4_·7H_2_O, 0.1 g/liter NiCl_2_·6H_2_O, 0.01 g/liter H_3_BO_3_, 0.01 g/liter CuSO_4_·5H_2_O] followed by addition of 1% (vol/vol) sterile glycerol and 50 μg/ml sterile kanamycin. For culturing of *Candida* and *Saccharomyces* strains, RPMI 1640 medium (Sigma-Aldrich R6504) was used for liquid culturing, and Sabouraud dextrose agar (SDA; Difco 210950) was used for solid plating unless otherwise specified. For *Aspergillus* strains, RPMI 1640 medium was used for liquid culturing, and potato dextrose agar (PDA; Difco 213400) was used for solid plating unless otherwise specified. YMM medium (6.7 g/liter yeast nitrogen base without amino acids [BD Difco 291940], 20 g/liter glucose, 1× trace element solution) was used as minimal medium for fungal strains.

10.1128/mBio.03402-20.3 TABLE S1 Microorganisms, primers, and plasmids used in this study. Download Table S1, DOCX file, 0.1 MB. Copyright © 2021 Polidore et al. 2021 Polidore et al. This content is distributed under the terms of the Creative Commons Attribution 4.0 International license.

### Bioinformatic analyses of phosphonate metabolism in *P. ananatis* strains.

*P. ananatis* genomes were downloaded from the NCBI database, and a hidden Markov model (HMM) search was performed using HMMER v3.2.1 (70). The HMMs used for analysis were phosphoenolpyruvate phosphomutase (*pepM*, TIGR02320), phosphonoacetaldehyde hydrolase (*phnX*, TIGR01422), phosphonoacetate hydrolase (*phnA*, TIGR02335), 2-AEP transaminase (*phnW*, TIGR02326 and TIGR03301), phosphonopyruvate hydrolase (*palA*, TIGR02321), phosphonoacetaldehyde dehydrogenase (*phnY*, TIGR03250), HD-phosphohydrolase (*phnZ*, TIGR00277 and PF01966), and C-P lyase (*phnJ*, PF06007). Genomes with an HMM match were considered to have the associated metabolism. Genomes containing *pepM* were screened for the presence of the *hvr* locus by mapping them to the *P. ananatis* LMG 5342 gene cluster using Geneious Prime v2020.2.1 software. For *pepM* genes that did not correspond to the *hvr* operon, gene cluster boundaries around the *pepM* were deduced based on the presence of either flanking integrative and conjugative elements or genes that appeared to be in an operon together with *pepM*. BLASTP was used to assign functions to genes in the gene cluster based on homology to proteins of known function.

### Genome sequencing of *Pantoea* NRRL strains.

The genomes of strains *P. ananatis* NRRL B-14773 and *P. ananatis* B-133 (since renamed P. stewartii based on NCBI average nucleotide identity analyses) were sequenced for use in this study. High-molecular-weight genomic DNA was purified using a Qiagen DNeasy UltraClean microbial kit. Purified genomic DNA was prepared using Shotgun Flex DNA library prep and sequenced using the Illumina MiSeq v2 platform (250-nucleotide [nt] paired-end reads) by the Roy J. Carver Biotechnology Sequencing Center, University of Illinois at Urbana-Champaign (UIUC). Genome reads were trimmed using BBDuk software, assembled using SPAdes v3.14.1, and annotated using the RAST server (71, 72). Assembled reads were submitted to the NCBI whole-genome shotgun (WGS) database. This WGS project has been deposited at DDBJ/ENA/GenBank under the accession numbers JACETZ000000000 (for *P. stewartii* NRRL B-133) and JACEUA000000000 (for *P. ananatis* NRRL B-14773). The genomes were analyzed for phosphonate metabolism as described above.

### Genetic methods for making unmarked deletion mutations.

Approximately 1 kb of DNA upstream and downstream of the region to be deleted was cloned into pHC001A (see Table S2 for the complete list of plasmid constructs). The resulting plasmids were introduced into electrocompetent E. coli DH5α/λpir and maintained in LB medium + 50 μg/ml kanamycin (LB-Kan). Plasmid constructs were moved into the conjugation strain WM6026 via electroporation-mediated transformation and then transferred to *P. ananatis* recipients via conjugation. Conjugations were performed by streaking isolated colonies of donor and recipient together in small (2 cm by 2 cm) patches on agar-solidified LB medium containing 60 μM diaminopimelic acid (DAP) to allow growth of WM6026-derived donor strains, which are DAP auxotrophs. After overnight incubation at 30°C, the patches were picked and restreaked on LB-Kan without added DAP to select for *P. ananatis* recombinants that carry the deletion plasmids, which cannot replicate autonomously in *P. ananatis*, and inserted into the target locus by homologous recombination. Exconjugants were purified by streaking for isolated colonies on LB-Kan at 30°C and then by streaking on LB without antibiotics to allow for segregation of the integrated plasmid. Recombinants that had lost the integrated plasmid were then isolated by streaking on LB medium without NaCl containing 5% sucrose, which selects against the *sacB* gene encoded on the integrated deletion plasmid. Loss of the integrated plasmid was verified by showing that the purified recombinants were kanamycin sensitive. Finally, recombinants carrying the desired deletion were identified by PCR-based screening using primers described in Table S3.

### Introduction of IPTG-inducible Ptac system.

Plasmid construct, pAP01, was introduced into WM6026 by electroporation with selection on LB + 50 μg/ml kanamycin (LB-Kan). The plasmid was then transferred to *P. ananatis* MMG1988 as described in the preceding section. Recombinants that carry an integrated copy of pAP01 inserted into the *hvrA* gene were selected on LB-Kan. The resulting colonies were screened for the plasmid integration using primers for *aph* and *lacI^q^* as well as the Hvr-marker gene *hvrI* (see Table S1 for the primer list). The resulting strain, *P. ananatis* MMG2010, was maintained in LB-Kan to prevent loss of the integrated plasmid.

### NMR and MS.

The ^1H^NMR, ^13^C-NMR, and ^31^P-NMR spectra were recorded on an Agilent DD2 600 MHz spectrometer (600 MHz for ^1^H, 150 MHz for ^13^C, and 243 MHz for ^31^P). Samples were prepared in 20 to 100% D_2_O as the locking solvent. Quantitative ^31^P-NMR was performed using an internal standard of 0.5 mM dimethylphosphinate with addition of 0.9 mM EDTA, and acquisition was performed using 5× the T1 measurement (relaxation time) for the sample. Phosphonate peak integrals were calculated using MestReNova v11.0.1 software and normalized to the internal standard. Concentrations were calculated based on the ratio of the normalized phosphonate peak integrals to the known concentration of internal standard. Mass spectrometry (MS) was performed by the School of Chemical Sciences Mass Spectrometry Laboratory using a Waters Q-TOF Ultima electrospray ionization (ESI) instrument in which 10 μl of sample was injected at a concentration of 10 μg/ml in methanol.

### IPTG-induced expression of the *hvr* operon in *P. ananatis* MMG2010.

A frozen glycerol stock of *P. ananatis* MMG2010 was revived on LB + 50 μg/ml kanamycin and incubated at 30°C for 24 h. A single colony was then transferred to 5 ml of phosphonate induction medium (PIM). The culture was incubated at 30°C for 48 h, and then 0.5 ml of culture was transferred to 50 ml PIM and incubated at 30°C for 24 h. The next day, 8 ml of culture was transferred to each of four flasks containing 800 ml PIM medium plus 1 mM IPTG. These 800-ml cultures were incubated with shaking at 175 rpm at 30°C for 72 h. After growth, cultures were centrifuged at 8,000 rpm for 20 min to remove cells and debris, and the supernatant was concentrated by freeze-drying. Quantitative ^31^P-NMR analysis was performed on the concentrated supernatant aliquot to determine phosphonate production levels after adding dimethylphosphinate (0.5 mM final) as an internal standard.

### Purification of pantaphos and compound 2.

A 3.2-liter culture of *P. ananatis* MMG2010 was grown in PIM medium with 1 mM IPTG as described in the preceding section. After centrifugation to remove cells, the spent medium was freeze-dried, and the dried material was resuspended in 300 ml H_2_O. Then, 1,200 ml of 100% cold methanol was added for a final concentration of 75% methanol and incubated at –20°C overnight. Precipitated material was removed and saved using vacuum filtration with a Whatman grade 42 ashless filter, and the methanol-soluble fraction was dried to completion using initial rotary evaporation followed by freeze-drying. The 48.0 g of dried material obtained by this process (sample A) was saved for further purification. The precipitated material saved from the above-described methanol extraction was subjected to a secondary 75% methanol extraction as described. Then the methanol-soluble fraction was dried to completion using initial rotary evaporation followed by freeze-drying. The 1.60 g of dried material obtained by this process (sample B) was saved for further purification. Sample A was subjected to Fe^3+^-IMAC purification as follows: 10 g of Chelex resin (sodium form) was converted to the H^+^ form by incubation in 1 M HCl for 30 min, followed by washing with 5 column volumes (CV) of water. Next, the resin was charged with Fe^+3^ by resuspension in 100 ml of 300-mM FeCl_3_·6H_2_O for 1 h at 4°C followed by washing with 100 ml of 0.1% acetic acid and incubation in 100 ml of 0.1% acetic acid overnight at 4°C. Sample A was acidified using concentrated acetic acid to pH 3 and incubated with the Fe-IMAC resin at 4°C for 2 h. The solution was separated from the resin using a gravity column, and the flowthrough containing unbound phosphonic acids was saved (sample-flowthrough). Bound phosphonic acids were eluted from the Fe-IMAC resin using a gradient of 100 ml NH_4_HCO_3_ (1, 5, 25, 50, 100, 250, 500, and 1,000 mM), and fractions were collected and neutralized to neutral pH using acetic acid. The eight fractions were concentrated using initial rotary evaporation followed by freeze-drying. Sample-flowthrough, containing any unbound phosphonic acids, obtained from the above-described process was combined with sample B and subjected to a second round of Fe-IMAC purification as described above. Fractions were concentrated using initial rotary evaporation followed by freeze-drying. From each of the Fe-IMAC purifications, the fractions were combined in 2 to 3 ml H_2_O as follows: the 500 to 1,000 mM NH_4_HCO_3_ fractions (sample 1), the 1 to 5 mM NH_4_HCO_3_ fractions (sample 2), and the 25 to 50, 100, or 250 mM NH_4_HCO_3_ fractions (sample 3). Samples 1, 2, and 3 were separately concentrated and dried via freeze-drying to obtain 45.1 mg, 30.6 mg, and 6.9 mg of dried material, respectively. Quantitative phosphorus NMR, based on a 0.5-mM dimethylphosphinate standard reference, and purity assessment using proton NMR were performed for each sample. Samples 1 and 3 contained 0.551 mmol and 0.215 mmol phosphonate, respectively. However, sample 2 was found to contain only 0.0165 mmol phosphonate and included residual phosphates not present in samples 1 and 3 and, therefore, was not used for further purification steps.

Sample 1 was subjected to further purification using a Teledyne ISCO CombiFlash RF+ UV-Vis system using a RediSep SAX anion exchange resin. Sample 1 was lyophilized and then reconstituted in 75% methanol. Insoluble material was removed by centrifugation, resuspended in 1 ml of 100% D_2_O, and examined by ^31^P-NMR. Samples containing residual phosphonates were subjected to additional cycles of drying and 75% methanol until all phosphonate compounds were solubilized. The methanol-soluble fractions were then pooled and subjected to CombiFlash purification. To do this, 5.7 g RediSep SAX column was equilibrated with 20 column volumes (CV) of 5% NH_4_OH in H_2_O, followed by 20 CV of H_2_O and then 20 CV of 90% methanol. A 1-ml sample from the methanol-soluble fractions was loaded onto the column via direct injection followed by 3.3 min 100% A (90% methanol), linear gradient to 100% B (5% NH_4_OH in H_2_O) over 15 min then 100% B for 6 min, followed by 3.5 min 100% A at an 18-ml/min flow rate. Fractions were monitored using UV 250 nm and 210 nm, and fractions showing absorbance at either wavelength were combined and analyzed by ^31^P-NMR. Fractions containing the *δ_P_* 18 and 15 ppm phosphorus chemical shifts were saved and dried via rotary evaporation followed by freeze-drying.

Sample 3 and the Combiflash-purified sample 1 were then subjected separately to high-pressure liquid chromatography (HPLC) purification. Dried samples were reconstituted in 1 ml H_2_O, and then 50 to 100 μl of sample was diluted in solvent B (90% acetonitrile + 10 mM NH_4_HCO_3_ at pH 9.20) for a final concentration of 75% solvent B. Samples were then filtered through a 0.45-μm filter and purified using Atlantis HILIC Silica column (10 × 250 mm^2^, 5 μm particle size) using gradient elution. Chromatography was performed at a flow rate of 4 ml/min using H_2_O + 10 mM NH_4_HCO_3_ at pH 8.50 (solvent A) and 90% acetonitrile + 10 mM NH_4_CO_3_ at pH 9.20 (solvent B). The gradient performed was as follows: 8 min at 90% solvent B, followed by a linear gradient to 70% solvent B over 20 min, then 50% solvent B over 1 min, hold at 50% solvent B over 8 min, and then back to 90% solvent B over 1 min, followed by hold at 90% solvent B for 8 min. Fractions were collected and monitored for UV absorption at 210 and 250 nm. Fractions that absorbed at these wavelengths were combined and dried via rotary evaporation and analyzed using phosphorus NMR. Fractions were obtained that contained a pure phosphonate compound with a *δ_P_* 15 ppm chemical shift (corresponding to pantaphos), and fractions were obtained containing a purified phosphonate compound with a *δ_P_* 18 ppm chemical shift (corresponding to compound 2). Additional fractions were obtained that contained a mixture of pantaphos and compound 2. Purified compounds were dried and saved at 4°C for MS and NMR structural analyses as described above.

### Onion bioactivity assays.

Yellow onions purchased form the local market were surface sterilized in a laminar flow biosafety hood as follows. First, the outermost layers with any damage or browning were removed and discarded, followed by soaking for 10 min in 10% bleach. The onions were then rinsed three times with sterile distilled water (dH_2_O), followed by soaking in 70% ethanol (EtOH) for an additional 10 min, and then rinsed four times with sterile dH_2_O. The onions were left in a biosafety laminar flow hood until the water and ethanol were completely evaporated. For testing the virulence of microbial strains, the onions were inoculated by stabbing with a previously sterilized wooden toothpick dipped into a bacterial cell suspension (1 × 10^3^ CFU/ml in 1× phosphate-buffered saline [PBS] buffer). Chemical complementation studies were performed by the addition of 100 μl of filter-sterilized sample into the hole created by the toothpick during inoculation. For testing the bioactivity of crude and purified chemical samples in the absence of bacteria, holes were punched in sterilized onions using sterile toothpicks followed by addition of 100 μl of a filter-sterilized sample. Following inoculation and/or treatment with filter-sterilized compounds, the onions were placed in Ziplock plastic bags and incubated at 30°C in the dark for the indicated number of days. Following incubation, the onions were sectioned across the site of inoculation/sample application to allow visual inspection of the center rot phenotype.

### Mustard seedling bioactivity testing.

All seed preparation was performed in a laminar flow biosafety hood. Burpee tendergreen mustard seeds were cleaned as follows: 10% bleach for 1 min, 3× rinse with sterile dH_2_O, 70% ethanol for 1 min, followed by 5× rinse with sterile dH_2_O. Washed seeds were transferred to a sterilized paper towel and placed inside a sterile container. Then, 5 ml of sterile tap water was added to the paper towel, and the container was incubated in the dark for 48 h to allow the seeds to germinate. Germinated seedlings were transferred to 24-well cell culture plates containing 1 ml of Murashige and Skoog agar (1% agar) per well. Then, 20 μl of an appropriate dilution of the filter-sterilized compounds being tested were added to each well to achieve the desired concentration. For negative controls, 20 μl of sterile dH_2_O was spotted onto the agar. The 24-well plates were incubated in a humidity-controlled growth room with a 16 h light cycle at 23°C for 1 week. Following incubation, plants were extracted from the growth agar by gentle pulling, which resulted in essentially agar free plants. Root length was measured immediately; dry weight was determined after drying for 24 h at 150°C. Statistical analysis was performed for each condition to determine significance using the standard Welch’s *t* test analysis from GraphPad Prism v8.4.1 software.

### Cell culture cytotoxicity screening.

The compounds were evaluated for their ability to kill cancer cell lines in culture, using HOS (osteosarcoma), ES-2 (ovarian cancer), HCT 116 (colon cancer), A549 (lung carcinoma), and A172 (glioma) cells. Human skin fibroblast cells (HFF-1) were also assessed. Cells were seeded (3,000 cells well^−1^ for ES-2, HCT 116, A549, and A172; 4,000 cells well^−1^ for HFF-1 and 2,500 cells well^−1^ for HOS) in a 96-well plate and allowed to attach overnight. Cells were treated with pantaphos in water. The concentrations of the tested compounds were 5 nM to 100 μM (1% water final; 100 μl well^−1^). Raptinal (50 μM) was used as a dead control. On each plate, five technical replicates per compound were performed. Then, 72 h posttreatment, cell viability was assessed using the alamarBlue method (http://www.bio-rad-antibodies.com/measuring-cytotoxicity-proliferation-spectrophotometry-fluorescence-alamarBlue.html). alamarBlue solution (10 μl of 440-μM resazurin in sterile 1× PBS) was added to each well, and the plate was incubated for 3 to 4 h. Conversion of alamarBlue was measured with a plate reader (SpectraMax M3; Molecular Devices) by fluorescence (excitation wavelength, 555 nm; emission wavelength, 585 nm; cutoff, 570 nm; autogain). The percentage death was determined by normalizing to water-treated cells and Raptinal-treated cells. For IC_50_ determination, the data were plotted as compound concentration versus dead cell percentage and fitted to a logistic dose-response curve using OriginPro 2019 (OriginLab). The data were generated in triplicate, and IC_50_ values are reported as the average of three separate experiments along with standard error of the mean SEM values.

### Antibacterial bioassays.

Susceptibility testing for the ESKAPE pathogens and Salmonella enterica LT2 was performed using a Kirby-Bauer method as outlined by the Clinical and Laboratory Standards Institute (CLSI) (73). For rich medium bioassays, Mueller-Hinton broth 2 (MH-2; Sigma-Aldrich; 90922) was used for all strains except that 1% brain heart infusion (BHI) was supplemented for Enterococcus faecalis ATCC 19433. For minimal medium bioassays, glucose-MOPS minimal medium was used. Briefly, overnight bacterial cultures were subcultured and inoculated into 5 ml top agar (0.7% agar) for a final concentration of 5 × 10^5^ CFU/ml. Then, 20 μl of a compound 2-fold dilution series (200, 100, 50, 25, 12.5, and 6.25 μM in water) was spotted on 6-mm diameter blank paper discs (BD BBL; 231039). Control discs received 20 μl of 50 mg/ml kanamycin. Plates were incubated at 35°C for 20 to 24 h. The MIC was recorded as the lowest concentration of compound that resulted in a clear zone of inhibition around the disc. Sensitivity of the IPTG-inducible phosphonate uptake strain WM6242 to pantaphos was tested using a disk diffusion assay. Plates containing growth medium were overlaid with 5 ml of top agar (0.7% agar) inoculated with 100 μl (optical density at 600 nm _[OD600]_ of 0.8) of the phosphonate-specific E. coli indicator strain (WM6242) with or without addition of 1 mM IPTG. WM6242 is engineered with an IPTG-inducible, nonspecific phosphonate uptake system (*phnCDE*). After the seeded overlay solidified, 6-mm paper disks were spotted with 10 μl of a dilution series (200, 100, 50, 25, 12.5, and 6.25 μM in water) of pantaphos and applied to the plates, which were then incubated at 37°C for 24 h. Phosphonate-specific activity was queried by comparing the sensitivity to that of 200 μM kanamycin and 200 μM fosfomycin. The MIC was recorded as the lowest concentration of compound that resulted in a clear zone of inhibition around the disc.

### Fungicidal bioassays.

Methods for fungicide testing were adopted from the CLSI publication M27, *Reference Method for Broth Dilution Antifungal Susceptibility Testing of Yeasts* (74). Briefly, a –80°C fungal stock was streaked on the appropriate medium as indicated previously and incubated at 35°C for 24 to 48 h. For *Candida* and *Saccharomyces*, a single colony was picked and resuspended in 1 ml 1× PBS and then diluted in growth medium to a final concentration of 1 × 10^4^ CFU/ml. For *Aspergillus*, spores from the hyphal growth on the growth medium plate were resuspended by swirling 1 ml of 90% saline + 0.1% Tween 20 onto the lawn. This 1-ml yeast suspension was then diluted in growth medium to a 1 × 10^4^ CFU/ml final concentration. Then, 2 μl of compound was added to 198 μl of the yeast suspension in a sterile 96-well round-bottom plate. For positive controls, 2 μl of a stock solution of amphotericin B was added to a final concentration of 2 or 10 μM as specified. Then, 200 μl of untreated yeast suspension and an uninoculated medium were included as controls. The plate was incubated at 35°C for 24 to 48 h depending on the growth medium without shaking. The MIC was determined visually by finding the concentration of compound at which there was no visual difference between that concentration and the uninoculated media control.

### Identification and analysis of homologous Hvr biosynthetic gene clusters in bacteria.

NCBI tblastn was used to identify bacterial genomes that contain Hvr-like gene clusters. The query was constructed by concatenating the gene translations of *hvrA* to *hvrL*. Tblastn was used against the RefSeq Genomes and the RefSeq Representative Genomes databases using the “Organism” parameter of “Bacteria [taxid:2]” and the nonredundant nucleotide database. The results were filtered by 45% query coverage, which resulted in 185 unique bacterial strains. The GenBank files for each strain were downloaded, and homologous Hvr gene clusters were determined using MultiGeneBlast (75). Homologous Hvr biosynthetic gene clusters were organized by type based on gene arrangement within the cluster and the presence of additional gene functions.

10.1128/mBio.03402-20.2 DATA SET S2 Structure elucidation of compound 1 (pantaphos) and compound 2 (phosphonomethylmaleate). Download Data Set S2, PDF file, 1.4 MB. Copyright © 2021 Polidore et al. 2021 Polidore et al. This content is distributed under the terms of the Creative Commons Attribution 4.0 International license.
