# Supplementary material for: A Phosphonate Natural Product Made by Pantoea ananatis is Necessary and Sufficient for the Hallmark Lesions of Onion Center Rot
Source: mBio. 2021 Feb 2;12(1):e03402-20. doi: 10.1128/mBio.03402-20 (PMC7858074; doi:10.1128/mBio.03402-20)
Supplement: TABLE S1 [file mBio.03402-20-st001.docx]

**Table S1. Microorganisms, primers and plasmids used in this study.**

| **Strain** | **Genotype/**  **Construction** | | **Source/**  **Reference** | |
| --- | --- | --- | --- | --- |
| *Escherichia coli* |  | |  | |
| DH5α/ *λpir* | *F- endA1 glnV44 thi-1 recA1 relA1 gyrA96 deoR nupG Φ80dlacZ* Δ*M15* Δ*(lacZYA-argF)U169, hsdR17(rK- mK^+^), λpir* | | (1, 2) | |
| WM6026 | *lacI^q^*, *rrnB3*, Δ*lacZ*4787, *hsdR*514, Δ*araBAD*567, Δ*rhaBAD*568, *rph-1*, *attλ*::pAE12(Δ*ori*R6K-*cat*::Frt5), Δ*endA*::Frt,*uidA*(ΔMluI)::*pir*,*att*HK::pJK1006 Δ(*ori*R6K-*cat*::Frt5;*trfA*::Frt) | | (3) | |
| WM6242 | *lacI^q^, rrnB3,* Δ*(lacZ4787), hsdR514, attP22(EcoB),* Δ*(araBAD)567,* Δ*(rhaBAD)568, rph-1,* Δ*(phnC-P),* Δ*(phoA), HKattB::pJK077(*Δ*aadA-oriR6K), lambda-attB::pJK074(*Δ*cat-oriR6K)* | | (4) | |
| *Pantoea ananatis* | |  | |  |
| LMG 5342 | Native phosphonate producer | | ATCC #22920 | |
| B-133 | wild-type | | ARS NRRL | |
| B-14773 | wild-type | | ARS NRRL | |
| MMG1888 | Δ*pgb* Δ*hvr*; Markerless deletion in MMG1984 using allele exchange plasmid pAP04 | | This study | |
| MMG1904 | Δ*phn;* Markerless deletion in LMG 5342 using allele exchange plasmid pAP05 | | This study | |
| MMG1912 | Δ*hvr;* Markerless deletion in LMG 5342 using allele exchange plasmid pAP04 | | This study | |
| MMG1920 | Δ*phn* Δ*hvr;* Markerless deletion in MMG1904 using allele exchange plasmid pAP04 | | This study | |
| MMG1984 | Δ*pgb;* Markerless deletion in LMG 5342 using allele exchange plasmid pAP02 | | This study | |
| MMG1988 | Δ*pgb* Δ*phn;* Markerless deletion in MMG1904 using allele exchange plasmid pAP02 | | This study | |
| MMG2010 | Δ*phn* Δ*pgb hvr*::pAP01; pAP01 recombinant host for over-expression of Hvr-BGC | | This study | |
| MMG2012 | Δ*pgb* Δ*phn* Δ*hvr;* Markerless deletion in MMG1988 using allele exchange plasmid pAP04 | | This study | |
| *Enterococcus faecalis* ATCC 19433 | | | ATCC #19433 | |
| *Staphylococcus aureus* ATCC 29213 | | | ATCC #29213 | |
| *Klebsiella pneumoniae* ATCC 27736 | | | ATCC #27736 | |
| *Acinetobacter baumannii* ATCC 19606 | | | ATCC #19606 | |
| *Pseudomonas aeruginosa* PAO1 | | | Hergenrother Lab (UIUC) | |
| *Escherichia coli* ATCC 25922 | | | ATCC #25922 | |
| *Salmonella enterica* LT2 | | | ATCC #700720 | |
| *Candida albicans* SN250 | | | Burke Lab (UIUC) | |
| *Aspergillus fumigatus* 1163 | | | Burke Lab (UIUC) | |
| *Saccharomyces cerevisiae* X2180-1A | | | Imlay Lab (UIUC) | |

| **Plasmids** | **Features/Construction/Use** | **Source/**  **Reference** |
| --- | --- | --- |
| pAE4 | *oriT*, Apr^R^, λ*attP*, ΦC31 *int*, ΦC31*attP* | (4) |
| pAH56 | *uidAF*, λ*attP*, *ori*R6K, Kan^R^, *lacI^q^*, Ptac | (5) |
| pAP01 | *hvrA*, *oriT*, λ*attP*, *ori*R6K, Kan^R^, *lacI^q^*, Ptac; Gibson assembly with pAP10 PCR product (PCR primers pAP10-rev/for) and *P. ananatis* LMG 5342 *pepM* gene (PCR primers Hvr-PepM-rev/for); used for integrating Ptac promotor upstream of Hvr-BGC | This study |
| pAP02 | *sacB*, Amp^R^, *ori*R6K, T7_p_, Kan^R^, lac_p_; Gibson assembly with pHC001A-SacI/XhoI-digested plasmid and 1kb upstream (PCR primers Pgb-left-hArm_R/F) and downstream (PCR primers Pgb-right-hArm_R/F) homology fragments to Pgb gene cluster; used to make markerless deletion of Pgb gene cluster | This study |
| pAP04 | *sacB*, Amp^R^, *ori*R6K, T7_p_, Kan^R^, lac_p_; Gibson assembly with pHC001A-SacI/XhoI-digested plasmid and 1kb upstream (PCR primers Hvr-left-hArm_R/F) and downstream (PCR primers Hvr-right-hArm_R/F) homology fragments to Hvr gene cluster; used to make markerless deletion of Hvr gene cluster | This study |
| pAP05 | *sacB*, Amp^R^, *ori*R6K, T7_p_, Kan^R^, lac_p_; Gibson assembly with pHC001A-SacI/XhoI-digested plasmid and 1kb upstream (PCR primers Phn-left-hArm_R/F) and downstream (PCR primers Phn-right-hArm_R/F) homology fragments to Phn gene cluster; used to make markerless deletion of Phn gene cluster | This study |
| pAP10 | *uidAF*, *oriT*, λ*attP*, *ori*R6K, Kan^R^, *lacI^q^*, Ptac; Gibson assembly with pAH56-SalI-digested plasmid and the *oriT* PCR-fragment from pAE4 plasmid (PCR primers pAH56-pAE4oriT-rev/for); used as parent plasmid for construction of pAP01 | This study |
| pHC001A | *sacB*, Amp^R^, *ori*R6K, T7_p_, Kan^R^, lac_p_; used as parent plasmid for allele exchange plasmid construction | (6) |

| **Primer Name** | **Sequence (5’->3’)** | **Use** |
| --- | --- | --- |
| Pgb-int-rev | CTTGCTGCAGGTAGGGGT | PCR-based assay for detection of phosphocholine cytidylyltransferase gene (n.t. 2,701,405 -> 2,700,635)^a^ within Pgb gene cluster |
| Pgb-int-for | TCTATCCACGGCAAACCACT |  |
| dPgb-rev | TGATGGCCTGCAAGACGG | PCR-based assay for detection of the deletion of Pgb gene cluster |
| dPgb-for | TCTATCCACGGCAAACCACT |  |
| Phn-int-rev | CAGCGCAACAGACTGGGA | PCR-based assay for detection of alpha-D-ribose 1-methylphosphonate 5-triphosphate diphosphatase gene (*phnM;* n.t. 1,796,249 -> 1,797,385)^a^ within Phn gene cluster |
| Phn-int-for | CCCATTCCGCCATGAGCA |  |
| dPhn-rev | ACGGTAAGATTGGGCGCC | PCR-based assay for detection of the deletion of Phn gene cluster |
| dPhn-for | GGCCAACGATCGCGGATA |  |
| PANA_3283  812-833R | GCTGCTATCCCCGAGATAATGA | PCR-based assay for detection of MFS transporter gene (*hvrI;* 809,348 -> 810,584)***^a^*** within Hvr gene cluster; primer sequences taken from reference (7) |
| PANA_3283 64-85F | GCTGAAGGGATTCAGACGGTTA |  |
| dHvr-rev | TTACCGCCACCTTGCTGG | PCR-based assay for detection of the deletion of Hvr gene cluster |
| dHvr-for | TTTCGCCCGTTCCCCTTC |  |
| PANA-Hvr-pepM-rev | GCCGTCCTGCCATATCTCAA | Detection of Hvr marker gene; Phosphoenolypyruvate mutase gene (*hvrA*; n.t. 801,910 -> 802,764)^a^ |
| PANA-Hvr-pepM-for | TAACGGACTCAGCATCTCGC |  |
| pHC001A-MCS-rev | CCCGACCCGAAACACCAT | Sequencing primer |
| pHC001A-MCS-for | TGTTCGCCAGGCTCAAGG | Sequencing primer |
| Phn-left-hArm_R | ACTAAAGGGAACAAAAGCTGGAGCTCCGCCAAAAATCAGCTGTG | For pAP05 construction |
| Phn-left-hArm_F | GGATTTAATTGTGAAAGACTCTCCGCTCGTG |  |
| Phn-right-hArm_R | GCGGAGAGTCTTTCACAATTAAATCCTCACATCAGTAGAGG |  |
| Phn-right-hArm_F | CTGGATGATCCTCCAGCGGGGCCCCCCCTCGAGTTCATGGCGCGGCTTTCG |  |
| Pgb-left-hArm_R | ATTTTATTTATTTAAACGTTAAACAAGAAATTCATC | For pAP02 construction |
| Pgb-left-hArm_F | TCCTCCAGCGGGGCCCCCCCTCGAGTTGAAGCGGCTAACTTCC |  |
| Pgb-right-hArm_R | AGGGAACAAAAGCTGGAGCTCTTGTTTCATCCATCATACC |  |
| Pgb-right-hArm_F | TTAACGTTTAAATAAATAAAATTGCTTGTCTCATG |  |
| Hvr-left-hArm_R | CTGACGGATTTTACAAACGCAAAAACCCCCGCC | For pAP04 construction |
| Hvr-left-hArm_F | CTGGATGATCCTCCAGCGGGGCCCCCCCTCGAGTAATCGCCGCCCACGCCG |  |
| Hvr-right-hArm_R | ACTAAAGGGAACAAAAGCTGGAGCTCCCATCATTACGTTTATGCC |  |
| Hvr-right-hArm_F | GTTTTTGCGTTTGTAAAATCCGTCAGGTGCAC |  |
| SEQ-pAP-rev | ACTATGAGCACGTCGGCG | sequencing primer |
| SEQ-pAP-for | CGCACTCCCGTTCTGGAT | sequencing primer |
| aph-rev | CAGGATGAGGATCGTTTCGC | Kan^R^ marker gene detection |
| aph-for | TCGAACCCCAGAGTCCCG | Kan^R^ marker gene detection |
| pAP10-rev | ATGTATATCTCCTTCTTACAAGCTTGGC | For pAP01 construction |
| pAP10-for | TCTAGATGCACTCCACCGCTGATGACATCAG |  |
| Hvr-PepM-rev | ATGTCATCAGCGGTGGAGTGCATCTAGATTAAGGAATCAGTGAAATAATTTC |  |
| Hvr-PepM-for | AAGCTTGTAAGAAGGAGATATACATATGATCAAAAAACTTATTGCAG |  |
| pAH56-pAE4oriT-rev | CATGAGAATTAATTCCGGGGATCCGTCGACACTACCATCGGGGGCCATC | For pAP10 construction |
| pAH56-pAE4oriT-for | CTACAGCCTCGGGAATTGCTGCAGGTCGACTCTAGATGCACTCCACCGC |  |

^a^n.t.= nucleotide positions of the gene from *P. ananatis* LMG 5342 genome sequence.

**References:**

1. **Kolter RI, M.; Helinski, D. R.** 1978. Trans-complementation-dependent replication of a low molecular weight origin fragment from plasmid R6K. Cell **15:**1199-1208.

2. **Grant SGNJ, J.; Bloom, F. R.; Hanahan, D.** 1990. Differential plasmid rescue from transgenic mouse DNAs into *Escherichia coli* methlyation-restriction mutants. Proc Natl Acad Sci U S A **87:** 4645–4649.

3. **Blodgett JA, Thomas PM, Li G, Velasquez JE, van der Donk WA, Kelleher NL, Metcalf WW.** 2007. Unusual transformations in the biosynthesis of the antibiotic phosphinothricin tripeptide. Nat Chem Biol **3:**480-485.

4. **Eliot AC, Griffin BM, Thomas PM, Johannes TW, Kelleher NL, Zhao H, Metcalf WW.** 2008. Cloning, expression, and biochemical characterization of *Streptomyces rubellomurinus* genes required for biosynthesis of antimalarial compound FR900098. Chem Biol **15:**765-770.

5. **Haldimann A, Wanner BL**. 2001. Conditional-replication, integration, excision, and retrieval plasmid-host systems for gene structure-function studies of bacteria. J Bacteriol **183:**6384-6393.

6. **Borisova SA, Christman HD, Metcalf ME, Zulkepli NA, Zhang JK, van der Donk WA, Metcalf WW.** 2011. Genetic and biochemical characterization of a pathway for the degradation of 2-aminoethylphosphonate in *Sinorhizobium meliloti* 1021. J Biol Chem **286:**22283-22290.

7. **Asselin JAE, Bonasera JM, Beer SV.** 2018. Center Rot of Onion (*Allium cepa*) Caused by *Pantoea ananatis* Requires pepM, a Predicted Phosphonate-Related Gene. Mol Plant Microbe Interact **31:**1291-1300.
