# Supplementary material for: A Phosphonate Natural Product Made by Pantoea ananatis is Necessary and Sufficient for the Hallmark Lesions of Onion Center Rot
Source: mBio. 2021 Feb 2;12(1):e03402-20. doi: 10.1128/mBio.03402-20 (PMC7858074; doi:10.1128/mBio.03402-20)
Supplement: DATA SET S2 [file mBio.03402-20-sd002.pdf]

Supplementary Dataset 2 for:

A phosphonate natural product made by *Pantoea ananatis* is necessary and sufficient for the hallmark lesions of onion center rot disease

Alexander L. A. Polidore, Lucia Furiassi, Paul J. Hergenrother, and William W. Metcalf

Structure elucidation of compound 1:

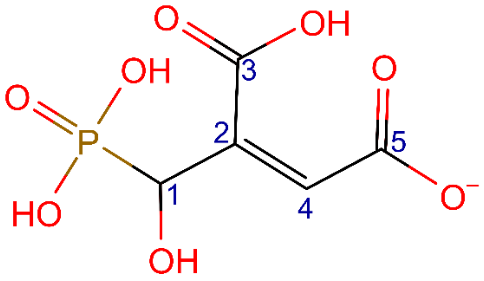

NMR spectral data are summarized in the main text (Fig. 3); NMR spectra and high-resolution mass-spectral data for compound 1 (pantaphos) are found at the end of this paragraph. MS chemical formulas and mass error was calculated using ChemCalc workspace (1). Compound 1 was isolated as a white, amorphous solid. Its molecular formula was deduced by negative mode HRMS (calcd. for $C_5H_6O_8P^{-1}$: 224.98058, observed *m/z* 224.9805 [Δppm 2.09]). Compound 1 was dissolved in 100% D_2O for NMR experiments. The ^{1}H-NMR spectrum for compound 1 revealed two signals at δ_H 4.31 and 5.91 ppm that appeared as doublets with coupling constants *J* of 15.3 Hz and 6.00 Hz, respectively. The large coupling constant *J* of 15.3 Hz is typical for protons bound to the adjacent carbon to a phosphorus atom in phosphonic acids (2). These protons were also correlated to the phosphorus atom of the compound at δ_P 15.40 ppm

in the ^{1}H-^{31}P HMBC analysis indicating close proximity (within 3-bond distance) to P. In addition, the downfield signal at δ_H 5.91 indicates a vinyl-carbon or alkene structure, which indicates that this signal corresponds to a single proton. The ^{13}C-NMR spectrum revealed signals at δ_C 71.00 (d, J=144.00 Hz), 142.98 (s), 174.62 (s), 126.50 (d, J=9.05 Hz), and 175.20 (s) ppm indicating compound 1 contains five carbons. The large coupling constant of the signal at δ_C 71.00 ppm suggests this carbon is bonded to the phosphorus atom as this splitting pattern has been observed for C-P bonding in other phosphonic acid compounds (2, 3). Therefore, this signal at δ_C 71.00 ppm is assigned as carbon position 1. None of the other carbon signals showed a typical C-P splitting pattern, therefore, the signals corresponding to these carbons must reflect carbon positions opposite the phosphonate moiety and adjacent to or nearby carbon 1. Proton-carbon HSQC and HMBC experiments revealed the coupling of proton at δ_H 4.31 ppm to the carbon at position 1 (δ_C 71.00 ppm), and was observed to correlate to the other carbons at δ_C 142.98, 174.62, and 175.20 ppm supporting the assignment of these carbons at positions adjacent to or nearby carbon 1. The carbon signal at δ_C 142.98 ppm has no splitting pattern and aligns with the chemical shifts predicted for vinyl compounds bound to an adjacent carboxylic acid and methyl group suggesting carbon position 2 assignment. The similar carbon signals at δ_C 174.62 and 175.20 ppm have no splitting pattern and align with the chemical shifts predicted for carboxylic acids suggesting assignment to carbon positions 3 or 5. However, the splitting of the carbon signal at δ_C 126.50 ppm (d, J=9.05 Hz) indicates the presence of an adjacent proton as the ^{13}C-NMR analysis was not performed with decoupling of ^{1}H. This is supported by the HSQC between this carbon and the proton at δ_H 5.91 ppm. These data fully support

the assignment of the carbon at δ_C 126.50 ppm to carbon position 4. Based on the proton-carbon HMBC between the protons at δ_H 4.31 and 5.91 ppm and the carbons at δ_C 142.98, 174.62, and 175.20 ppm, we were able to confirm assignment of carbons at δ_C 142.98, 174.62, and 175.20 ppm to positions 2, 3, and 5, respectively. Finally, after ^1H-^1H correlation analyses, it was determined that the protons at δ_H 4.31 and 5.91 ppm are arranged in a *cis* carbon-carbon double bond configuration. Based on the agreements between the MS data and these NMR assignments the compound structure is identified as *(E)*-2-(hydroxy(phosphono)methyl)-4-oxopent-2-enoate.

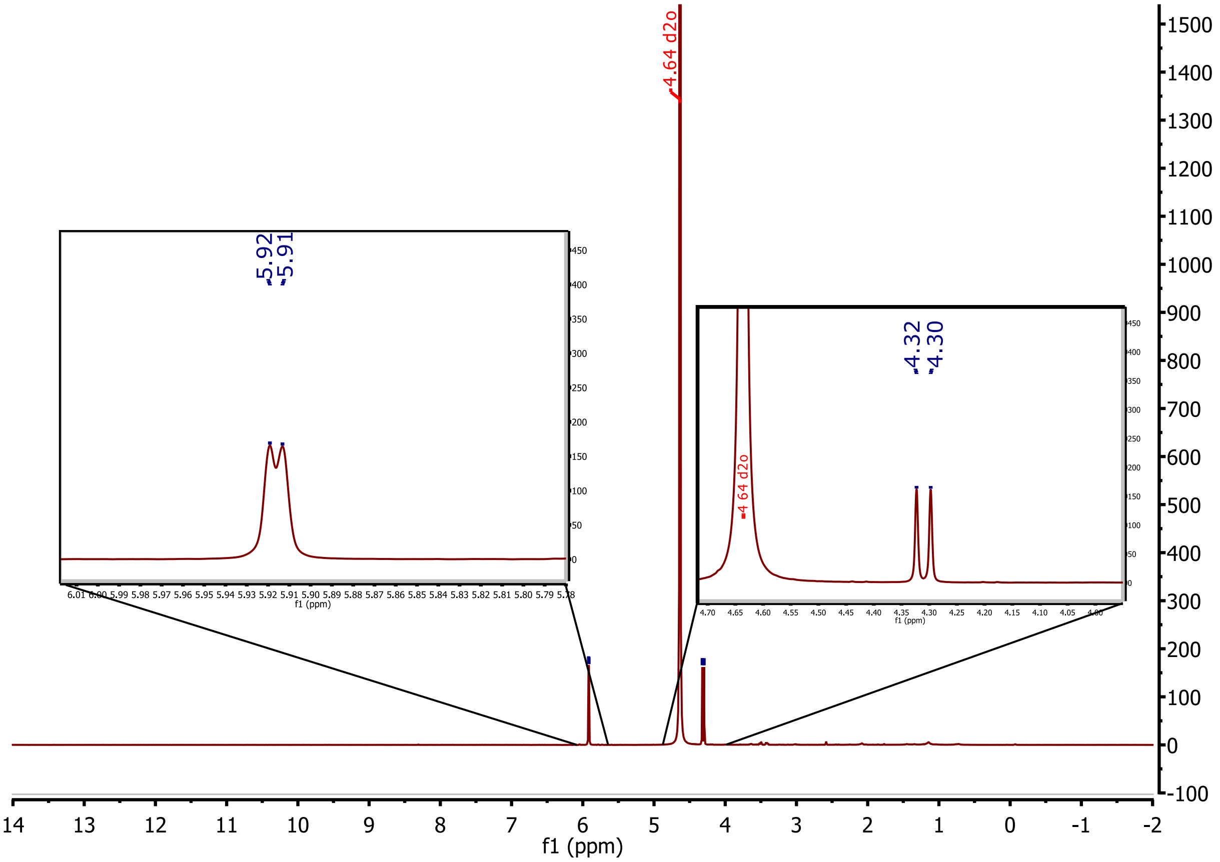

^{1}H NMR spectrum for purified pantaphos.

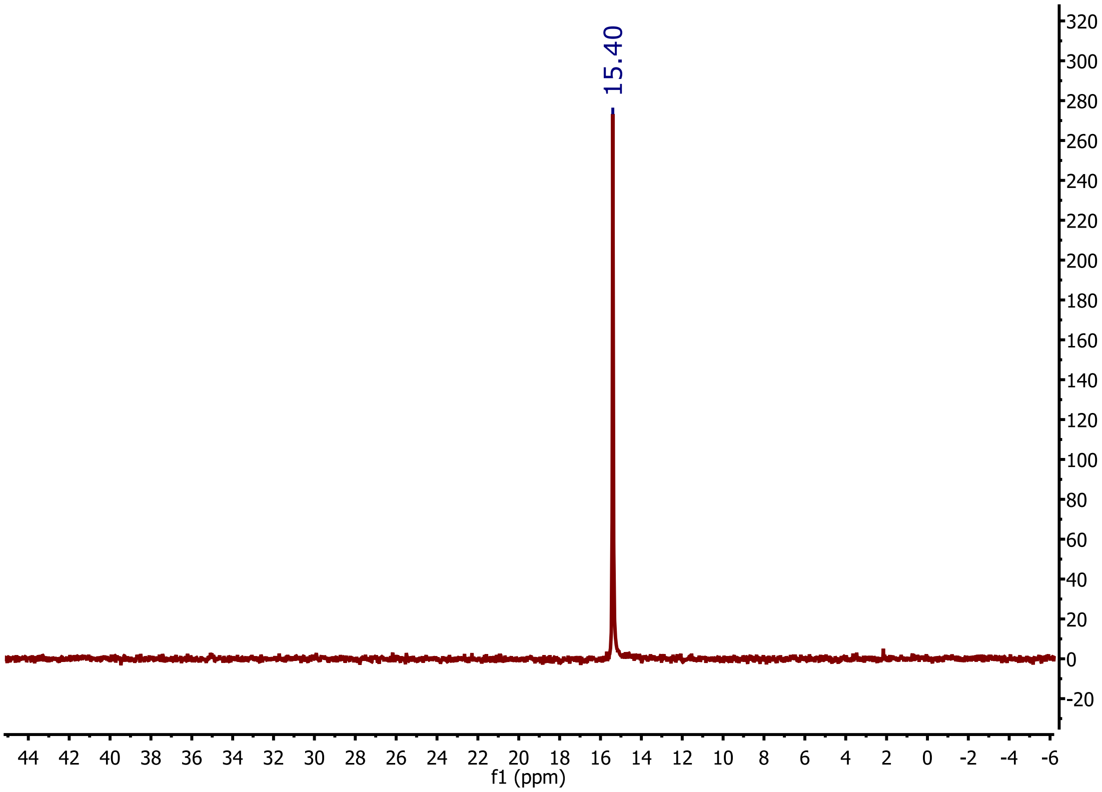

^{31}P NMR spectrum for purified compound 1 (pantaphos)

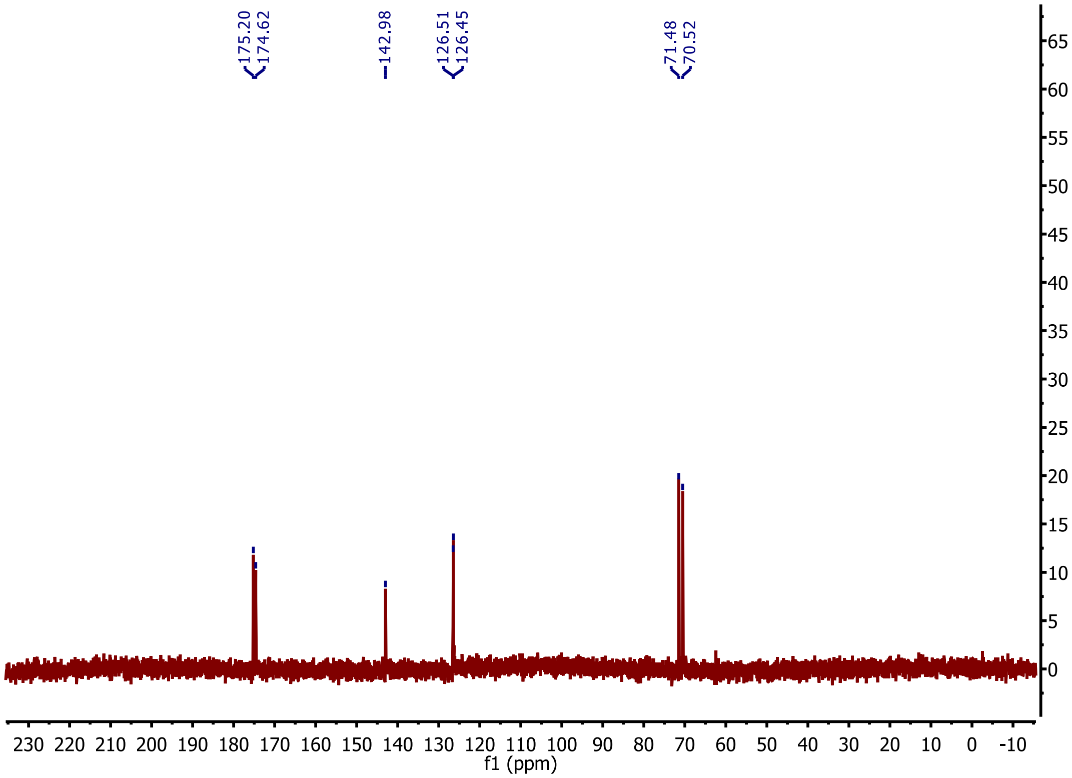

^{13}C NMR spectrum for purified compound 1 (pantaphos)

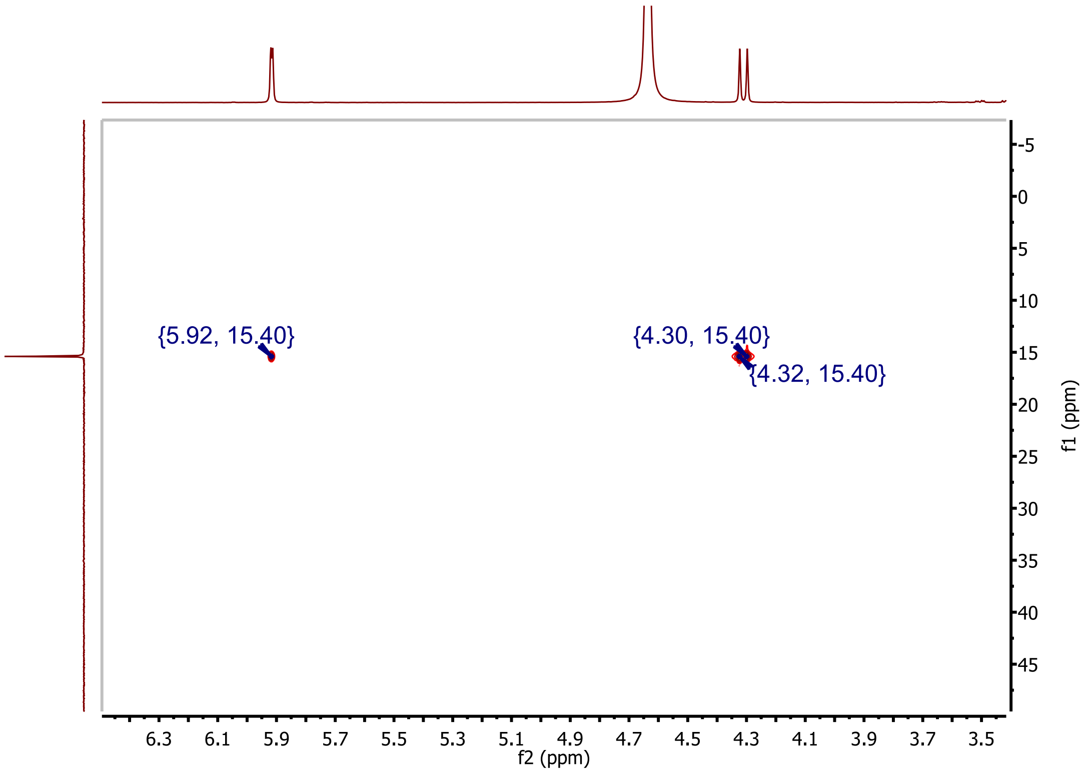

^{1}H-^{31}P HMBC spectrum for purified compound 1 (pantaphos)

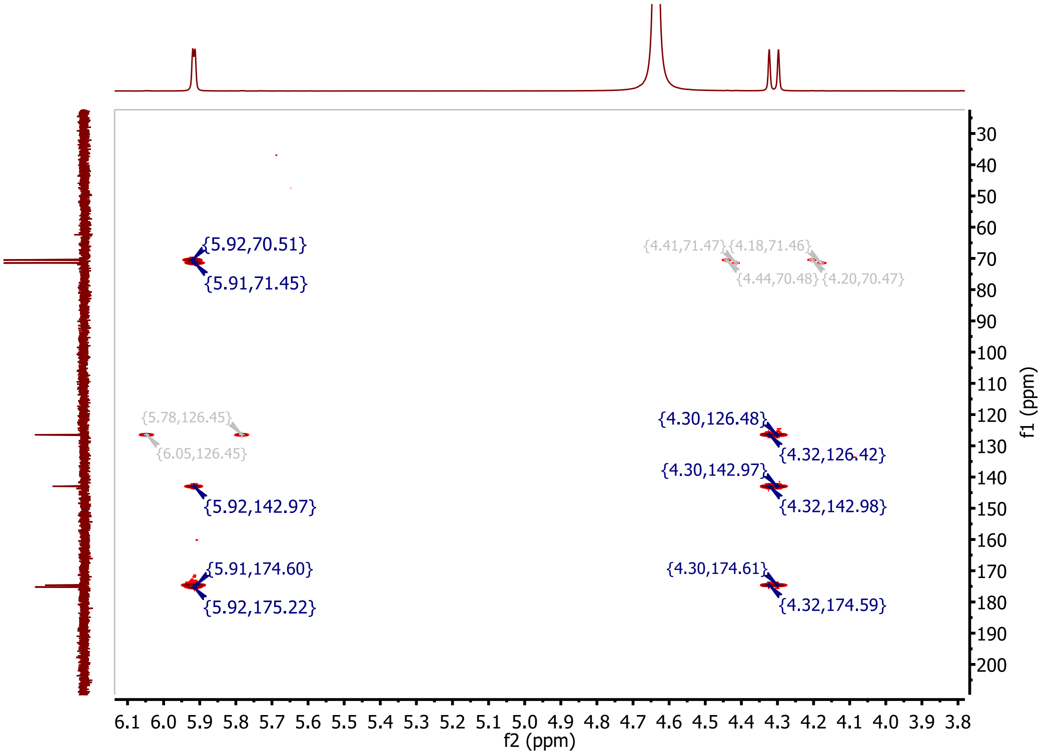

^{1}H-^{13}C HMBC spectrum for purified compound 1 (pantaphos). Greyed peaks indicate NMR signals caused by the strong spin coupling of the direct bonding between the carbon and its proton.

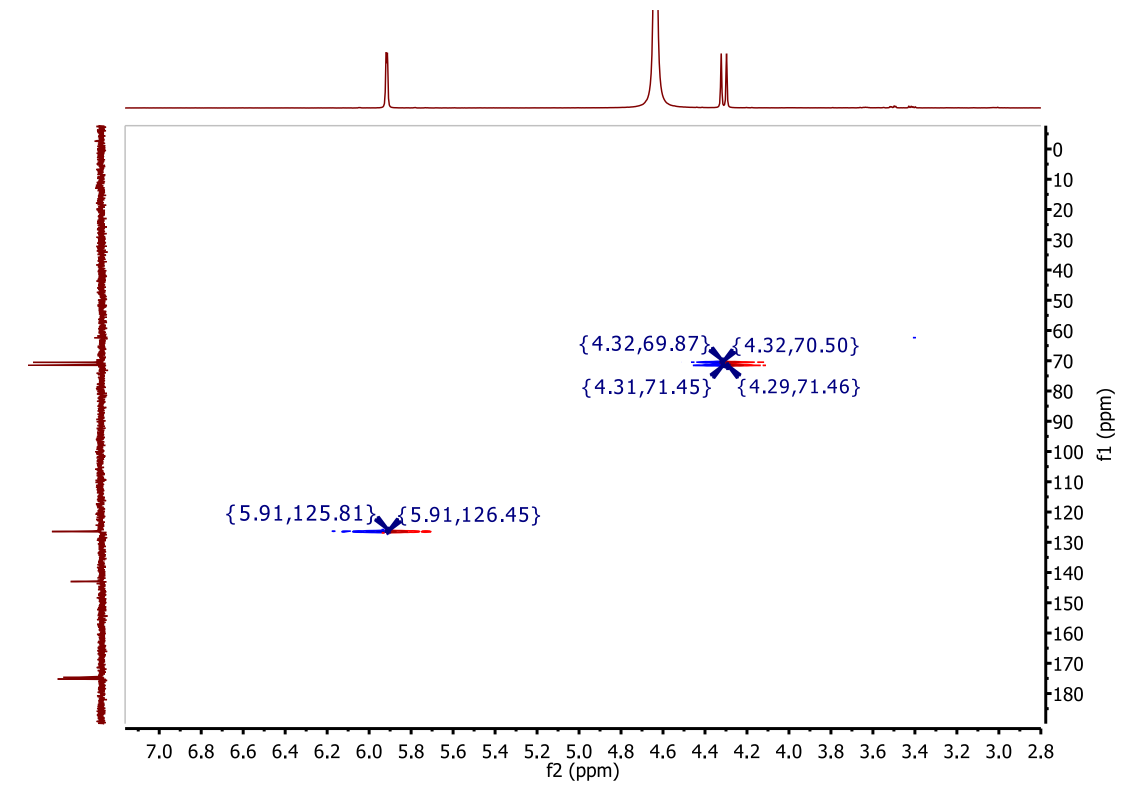

^{1}H-^{13}C **HSQC spectrum for purified compound 1 (pantaphos)**

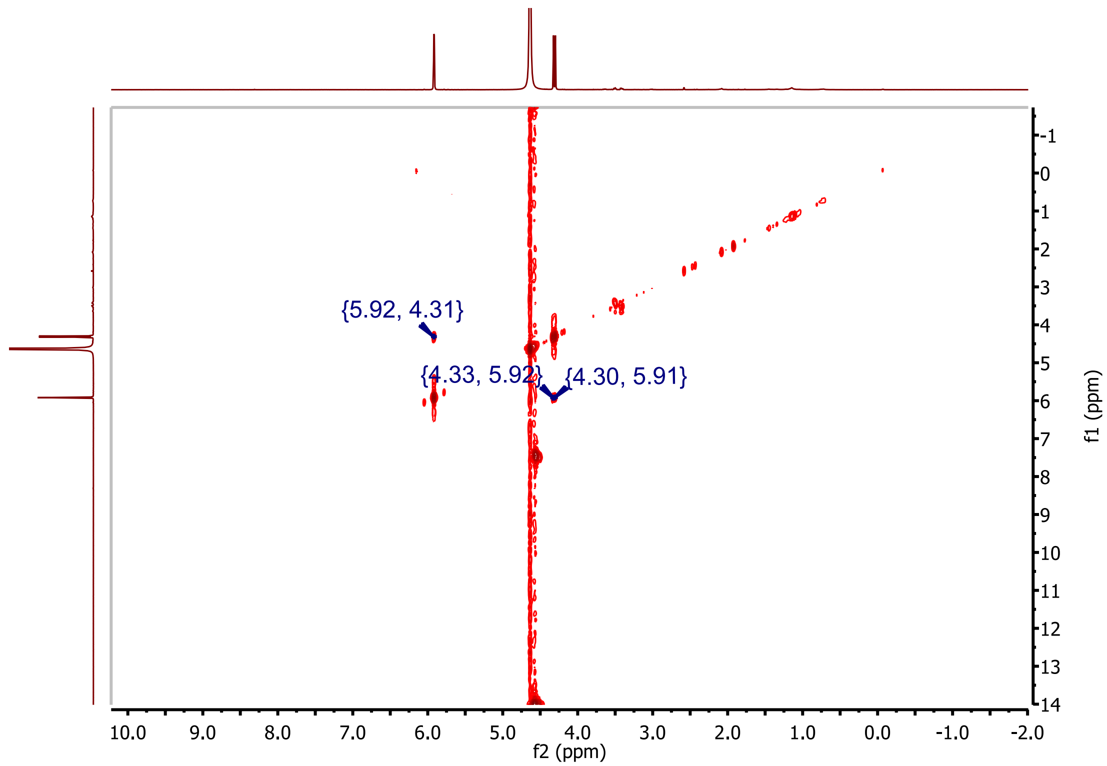

^{1}H-^{1}H **gCOSY spectrum for purified compound 1 (pantaphos)**

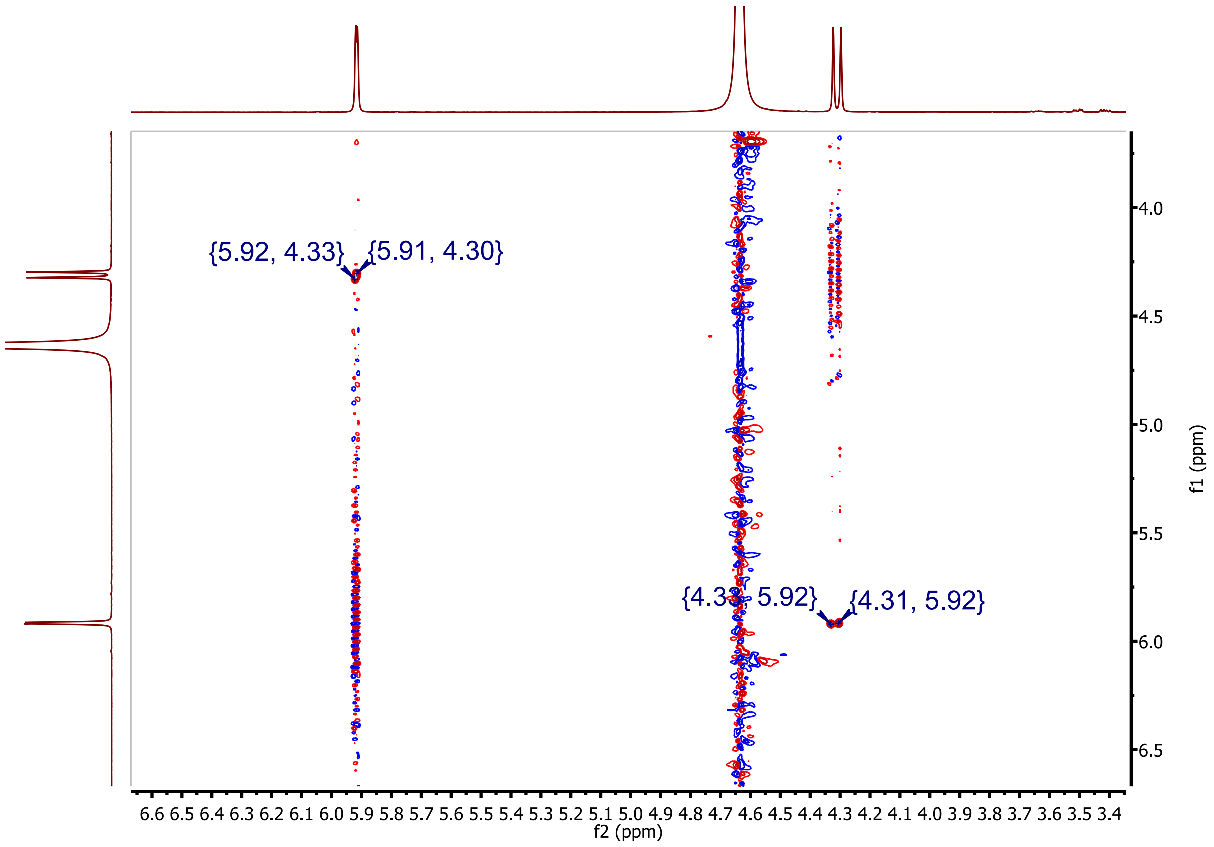

^{1}H-^{1}H NOSEY spectrum for purified compound 1 (pantaphos)

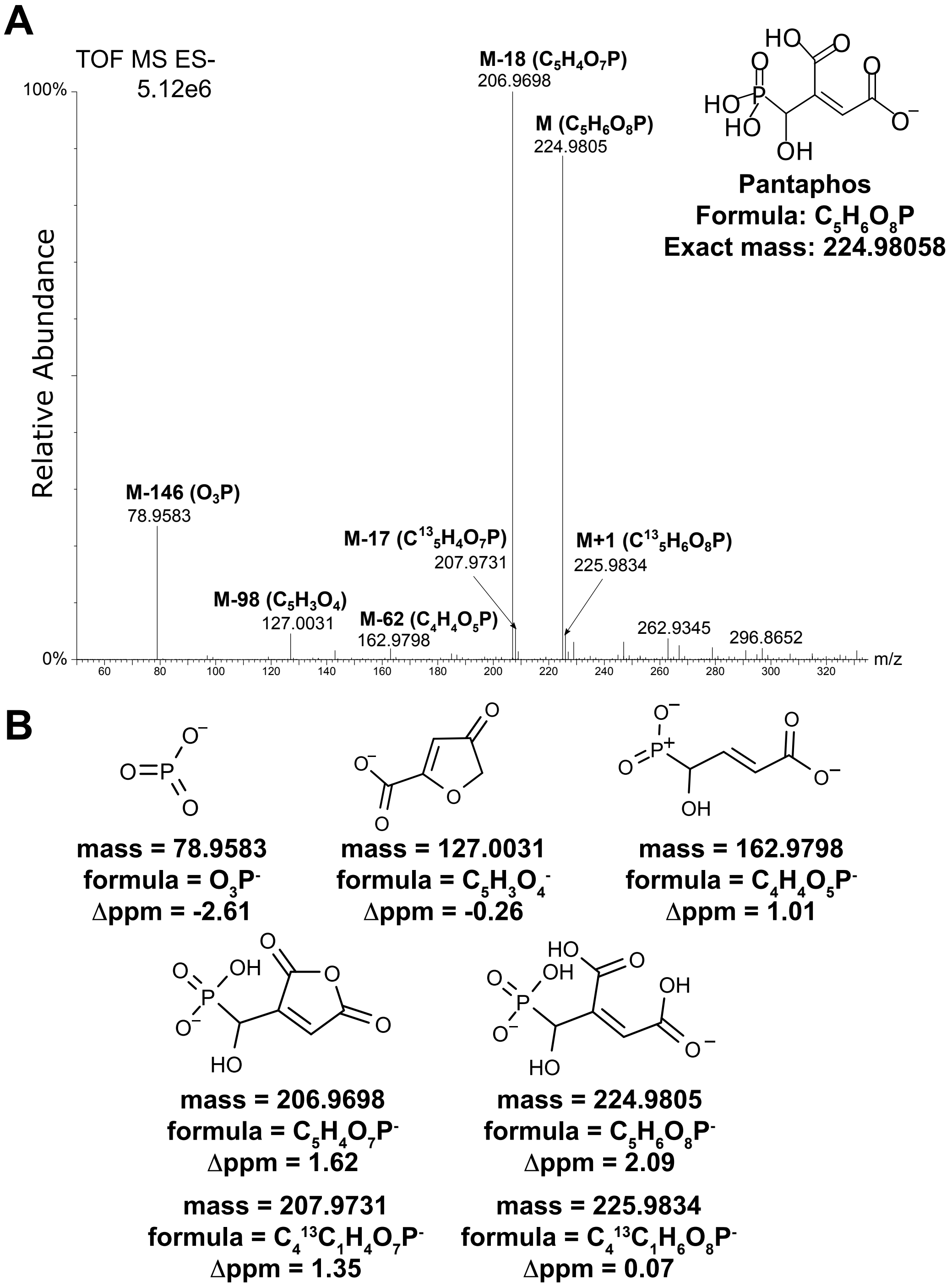

High-resolution TOF MS/ESI- spectrometry for compound 1 (pantaphos). High resolution mass spectrum for purified pantaphos (A) and the designated mass fragments with assigned chemical structures (B). The molecular ion was identified as *m/z* 224.9805 with chemical formula $C_5H_6O_8P^-$ corresponding to pantaphos.

Structure elucidation of compound 2:

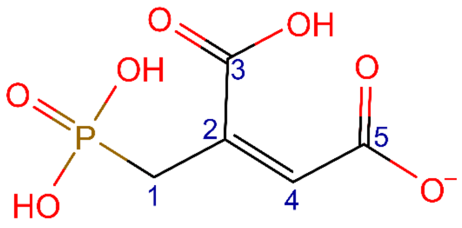

NMR spectral data are summarized in the main text (Fig. 3); NMR spectra and high-resolution mass-spectral data for compound 2 are found at the end of this paragraph. MS chemical formulas and mass error was calculated using ChemCalc workspace (1). Compound 2 was isolated as a white, amorphous solid. Its molecular formula was deduced by negative mode HRMS (calcd. for $C_5H_6O_7P^{-1}$: 208.98566, observed *m/z* 208.9851 [Δppm -0.07]; Fig. S23). Fractions containing pure compound 2 were dissolved in 100% D_2O and subjected to proton and phosphorus NMR analyses. For carbon NMR experiments, a sample containing trace amounts of compound 1 were used as there was not enough concentrated pure compound 2 to perform carbon-13 analyses. The ^{1}H-NMR spectrum for compound 2 revealed two signals at δ 5.71 (d, J=6.00 Hz) and 2.43 ppm (d, J=18.0 Hz). The large coupling constant *J* of 18.0 Hz is typical for protons bound to the adjacent carbon to a phosphorus atom in phosphonic acids (2). These protons were also correlated to the phosphorus atom of the compound at δ_P 18.46 ppm in the ^{1}H-^{31}P HMBC analysis indicating close proximity (within 3-bond distance) to P. In addition, the downfield signal at δ_H 5.71 indicates a vinyl-carbon or alkene structure, which indicates that this signal corresponds to a single proton. ^{13}C-NMR analysis revealed signals at δ_C 34.05 (d, J=123.0 Hz), δ 126.10 (d, J=10.60 Hz), δ 140.36 (s), δ 174.68 (s), and δ 177.02 (s) ppm associated with compound 2 indicating a

five-carbon molecule. The large coupling constant of the carbon signal at δ_C 34.05 ppm suggests this carbon is bonded to the phosphorus atom as this splitting pattern has been observed for C-P bonding in other phosphonic acid compounds (2, 3). Therefore, this signal at δ_C 34.05 ppm is assigned as carbon position 1. None of the other carbon signals showed a typical C-P splitting pattern, therefore, the signals corresponding to these carbons must reflect carbon positions opposite the phosphonate moiety and adjacent to or nearby carbon 1. Proton-carbon HSQC and HMBC experiments revealed the coupling of protons at δ_H 2.43 ppm to the carbon at position 1 (δ_C 34.05 ppm), and was observed to correlate to the other carbons at δ_C 140.36, 174.68, and 177.02 ppm supporting the assignment of these carbons at positions adjacent to or nearby carbon 1. The carbon signal at δ_C 140.36 ppm has no splitting pattern and aligns with the chemical shifts predicted for vinyl compounds bound to an adjacent carboxylic acid and methyl group suggesting carbon position 2 assignment. The similar carbon signals at δ_C 174.68 and 177.02 ppm have no splitting pattern and align with the chemical shifts predicted for carboxylic acids suggesting assignment to carbon positions 3 or 5. However, the splitting of the carbon signal at δ_C 126.10 ppm (d, J=10.60 Hz) indicates the presence of an adjacent proton as the ^{13}C-NMR analysis was not performed with decoupling of ^{1}H. This is supported by the HSQC between this carbon and the proton at δ_H 5.71 ppm. These data fully support the assignment of the carbon at δ_C 126.10 ppm to carbon position 4. Based on the proton-carbon HMBC between the protons at δ_H 2.43 and 5.71 ppm and the carbons at δ_C 140.36, 174.68, and 177.02 ppm, we were able to confirm assignment of carbons at δ_C 140.36, 174.68, and 177.02 ppm to positions 2, 3, and 5, respectively. Finally, after ^{1}H-^{1}H correlation analyses, it was determined that the

protons at δ_H 2.43 and 5.71 ppm are arranged in a *cis* carbon-carbon double bond configuration. Based on the agreements between the MS data and these NMR assignments the compound structure is identified as *(E)*-2-(phosphono)methyl)-4-oxopent-2-enoate.

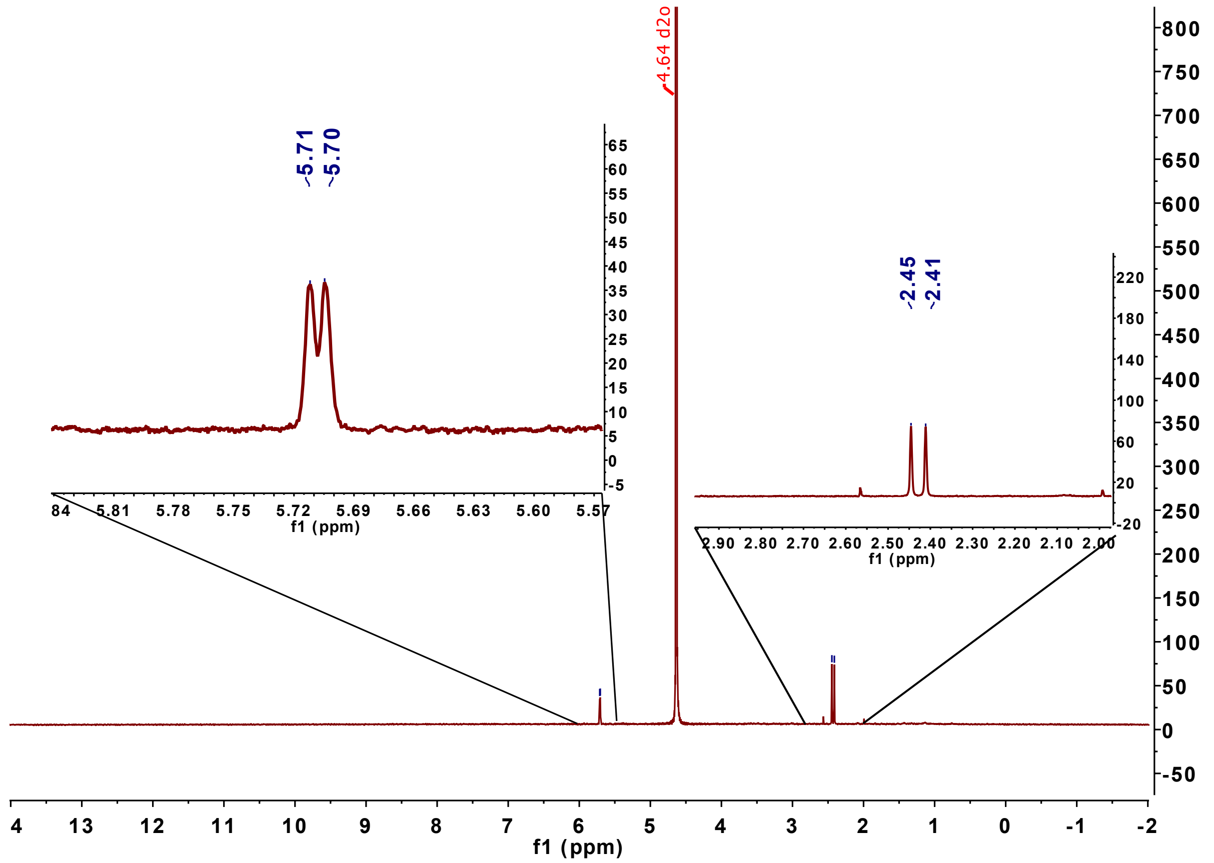

^{1}H NMR spectrum for purified compound 2.

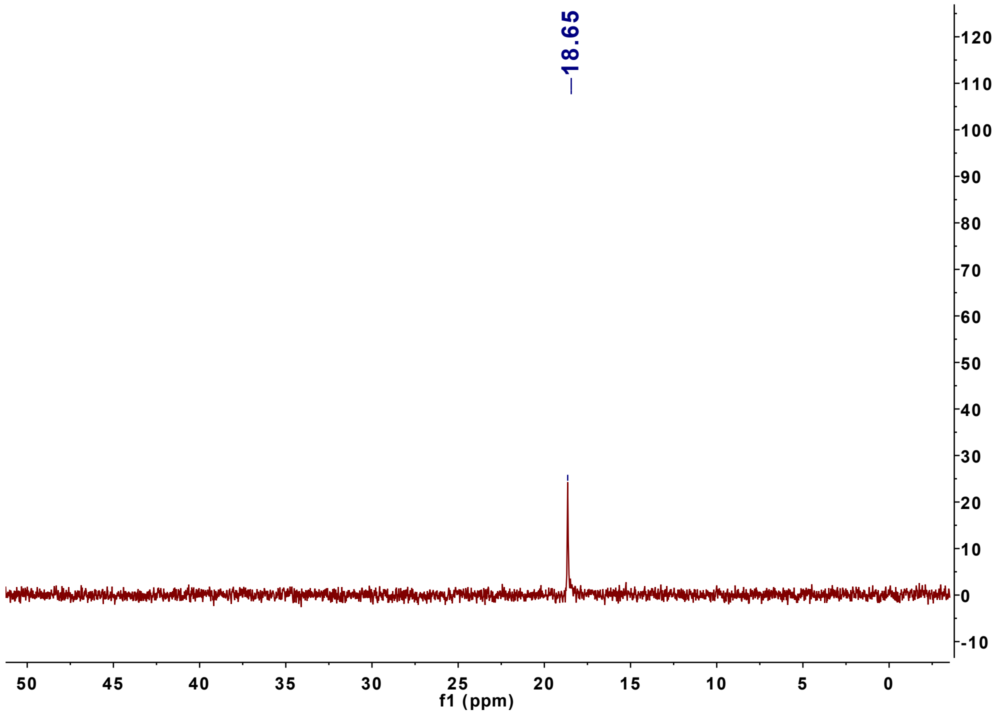

^{31}P NMR spectrum for purified compound 2.

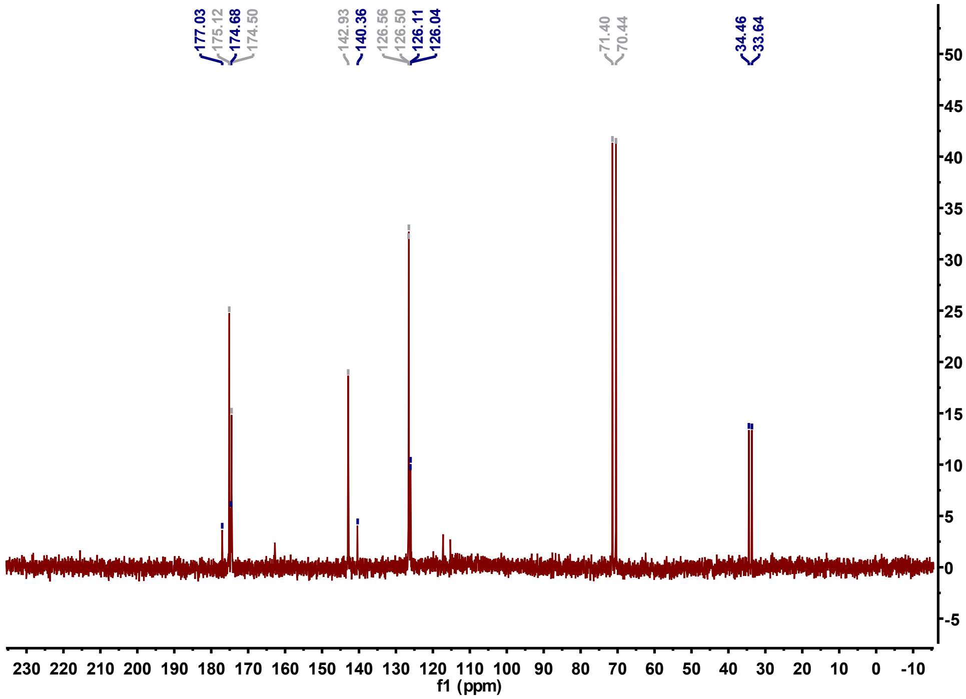

^{13}C NMR spectrum for partially purified compound 2. We were unable to isolate sufficient quanties of pure compound 2 to allow collection of HMBC spectra in a reasonable time-frame. Accordingly, we used a mixed fraction containing some amount of pantaphos for this analysis. Thus, the spectrum also includes signals that can be confidently assigned to pantaphos (shown in grey).

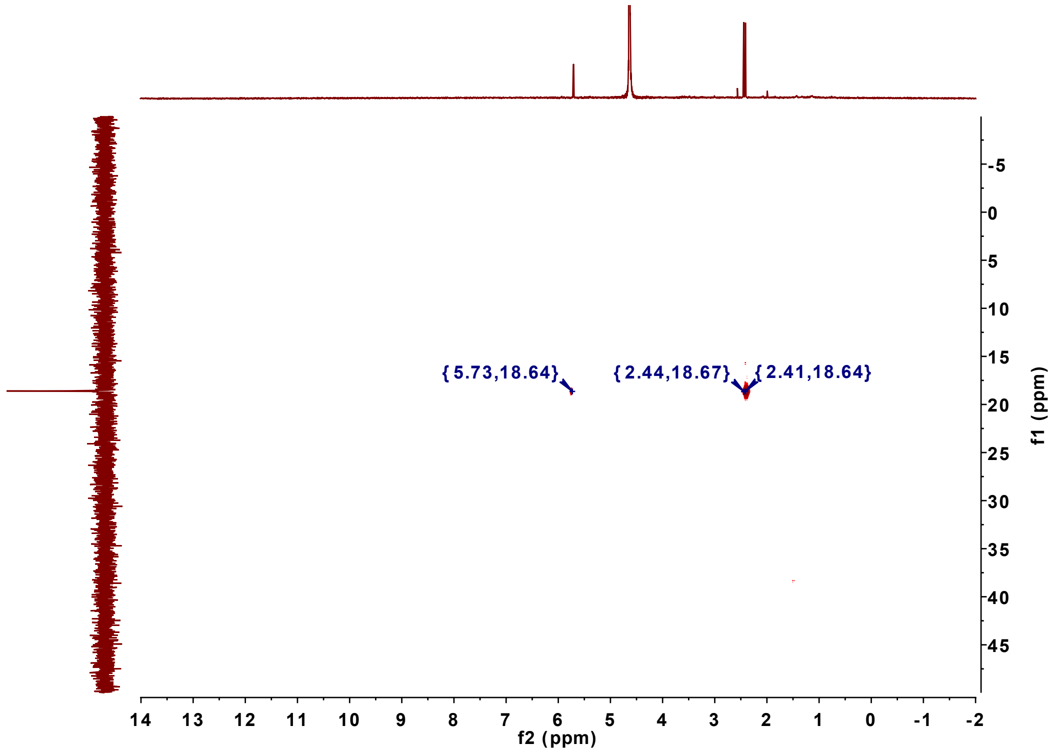

^{1}H-^{31}P HMBC spectrum for purified compound 2.

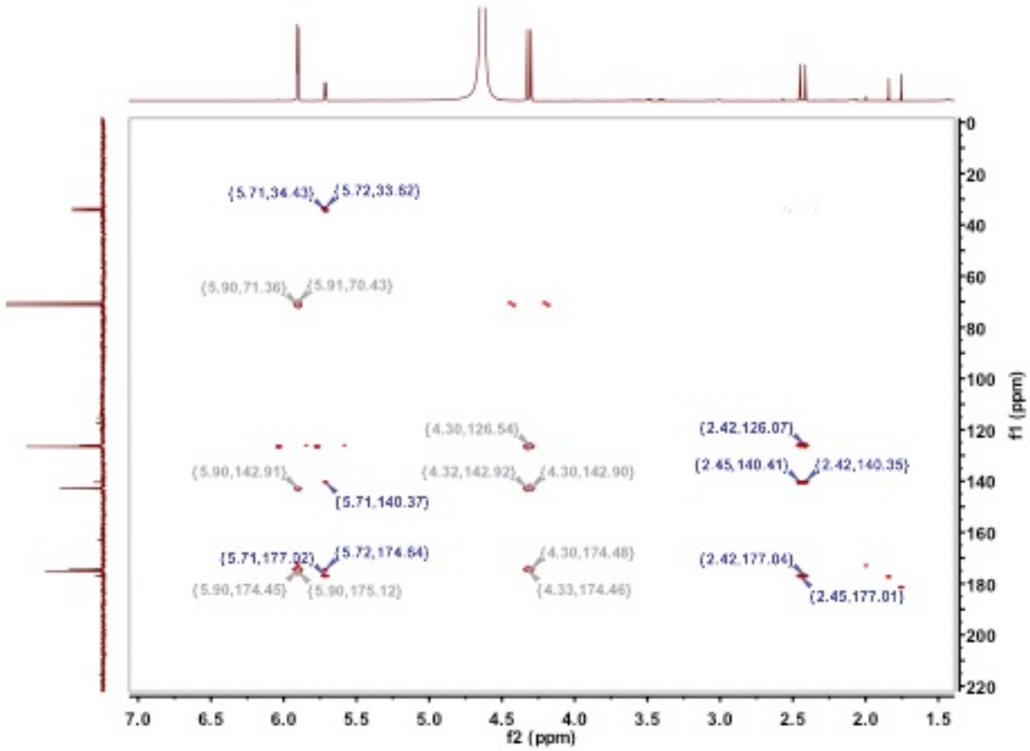

^{1}H-^{13}C HMBC spectrum for partially purified compound 2. We were unable to isolate sufficient quanties of pure compound 2 to allow collection of HMBC spectra in a reasonable time-frame. Accordingly, we used a mixed fraction containing some amount of pantaphos for this analysis. Thus, the spectrum also includes signals that can be confidently assigned to pantaphos (shown in grey).

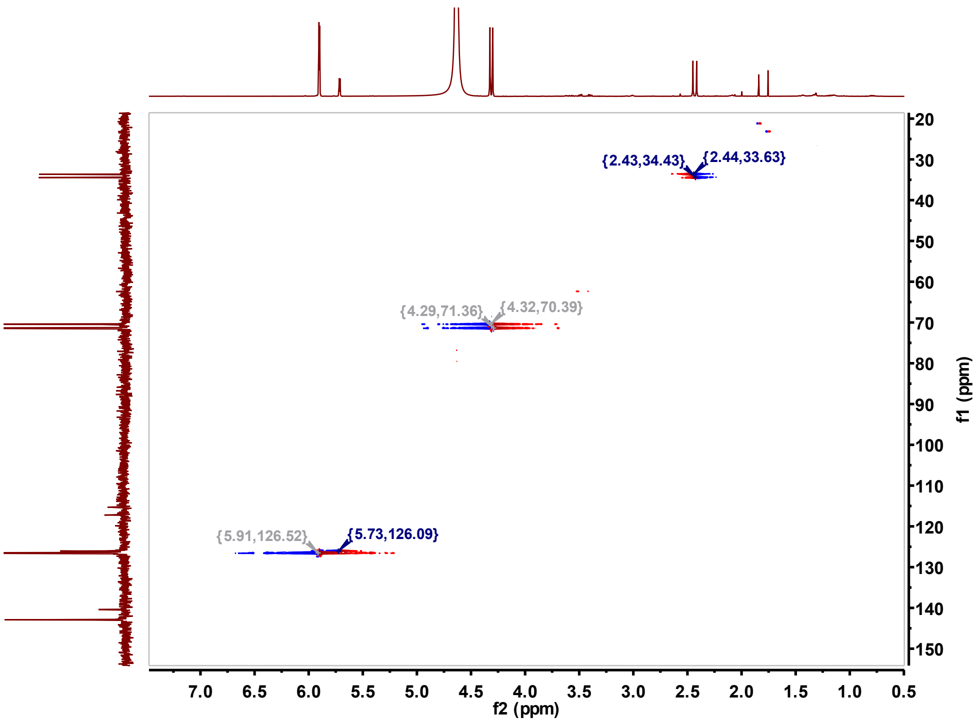

^{1}H-^{13}C HSQC spectrum for partially purified compound 2. We were unable to isolate sufficient quanties of pure compound 2 to allow collection of HSQC spectra in a reasonable time-frame. Accordingly, we used a mixed fraction containing some amount of pantaphos for this analysis. Thus, the spectrum also includes signals that can be confidently assigned to pantaphos (shown in grey).

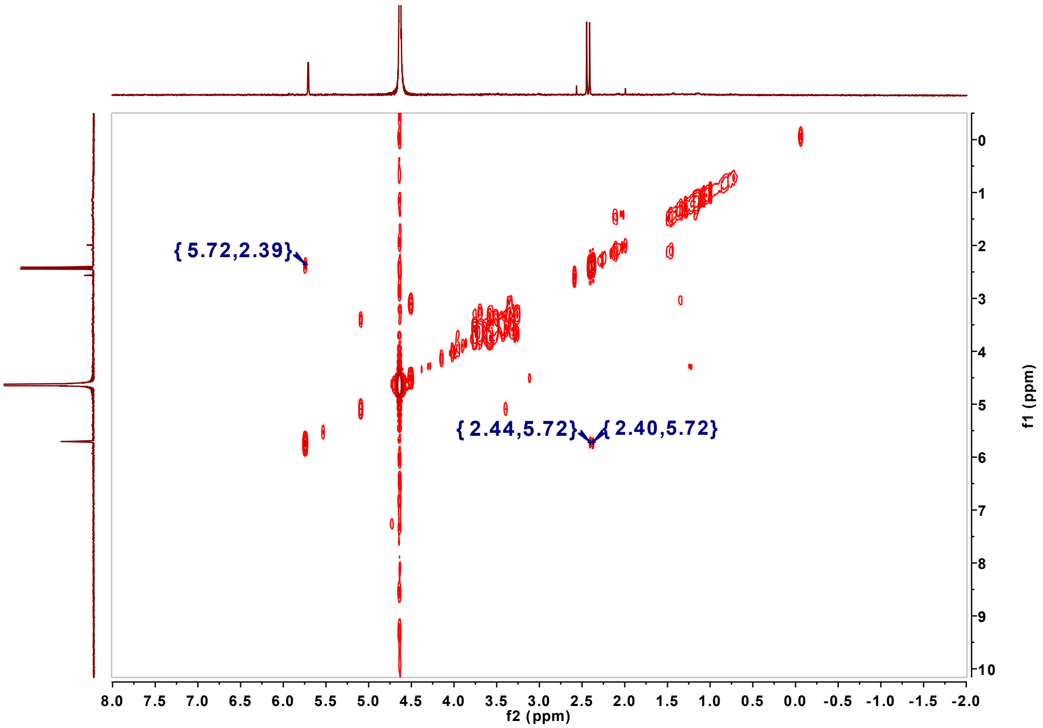

^{1}H-^{1}H gCOSY for pure compound 2.

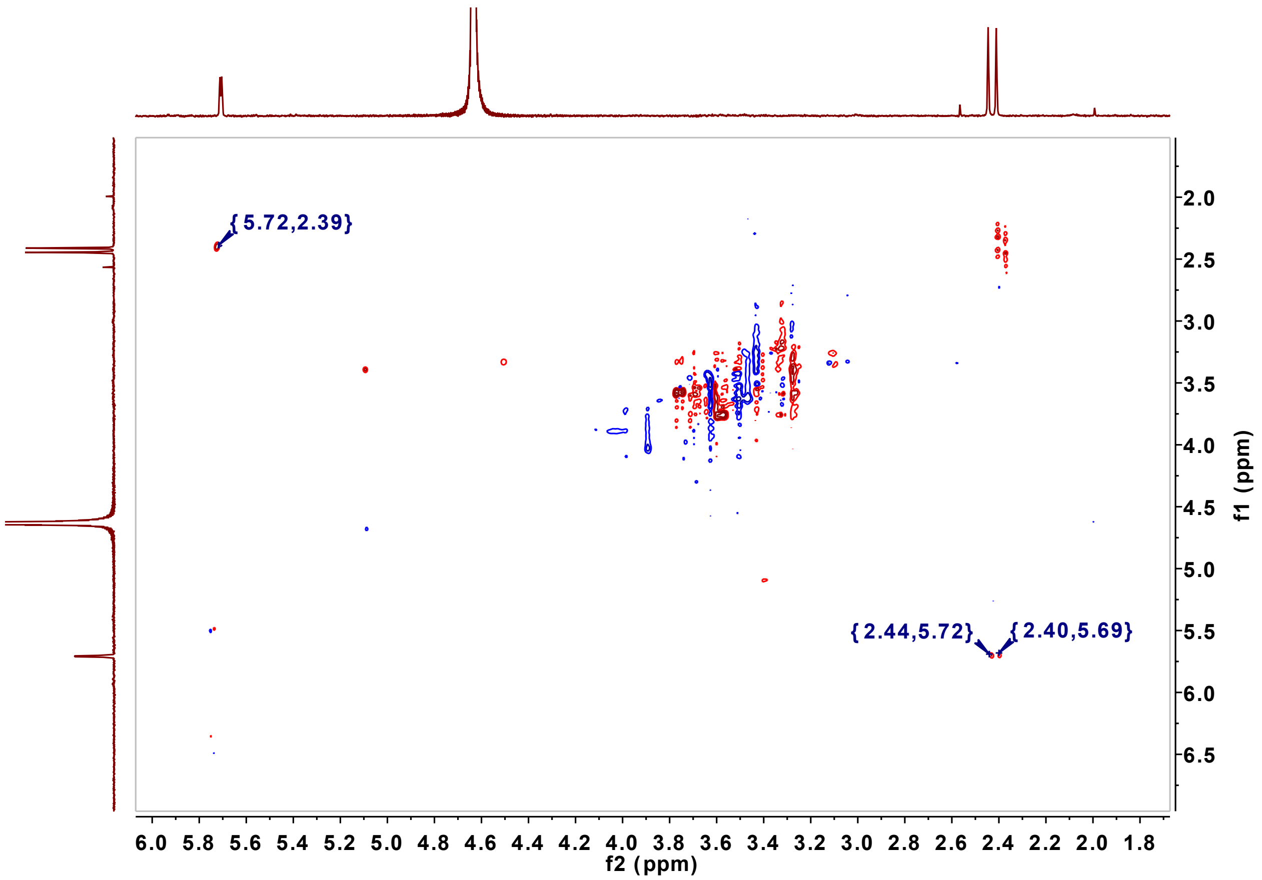

^{1}H-^{1}H NOSEY for pure compound 2

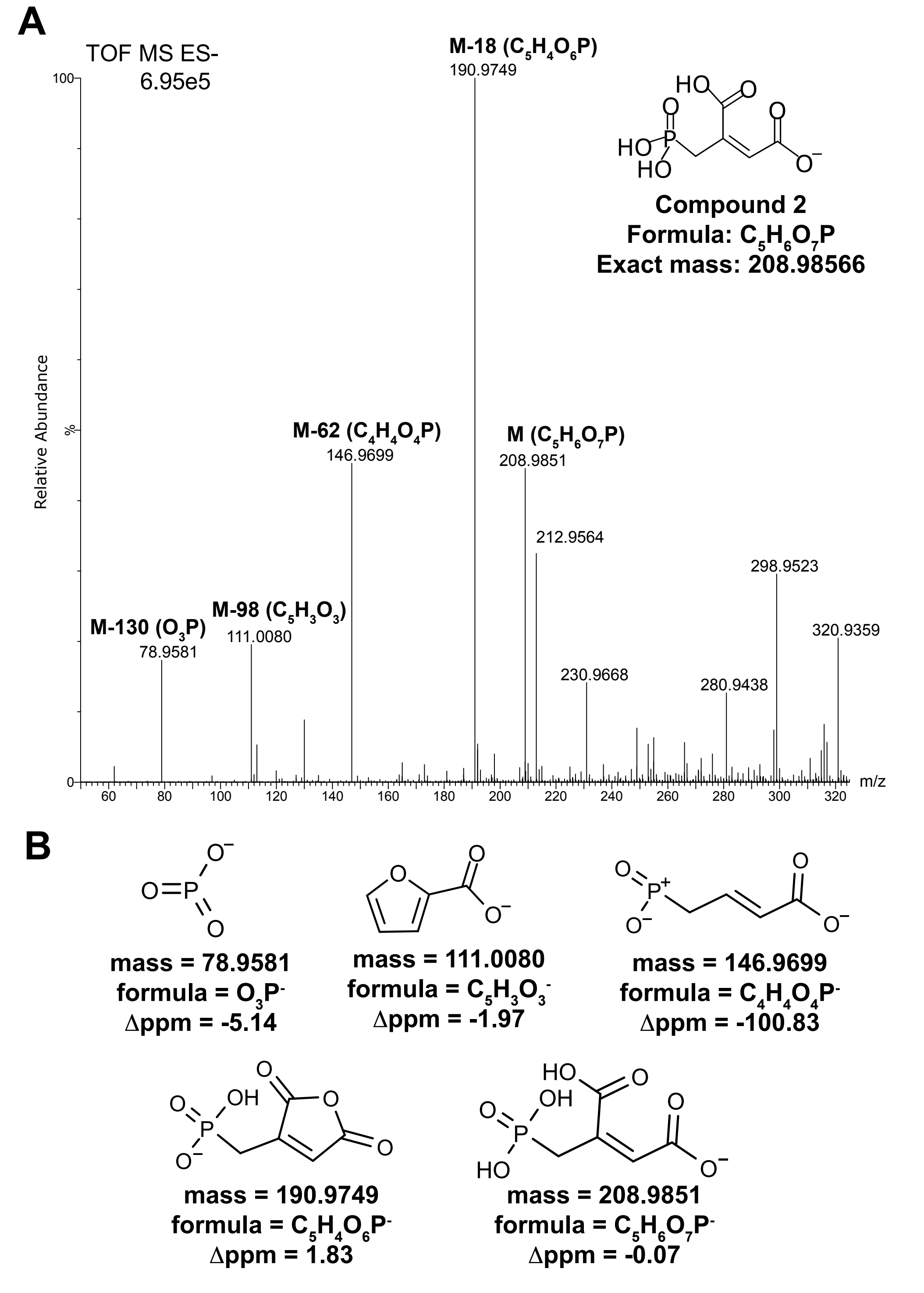

High-resolution TOF MS/ESI- spectrometry for compound 2. High resolution mass spectrum for purified compound 2 (A) and the designated mass fragments with assigned chemical structures (B). The molecular ion was identified as *m/z* 208.9851 with chemical formula $C_5H_4O_7P^-$ corresponding to compound 2 (2-phosphonomethylmaleate).

References

1. **Patiny L, Borel A.** 2013. ChemCalc: a building block for tomorrow's chemical infrastructure. J Chem Inf Model **53:**1223-1228.
2. **Cioni JP, Doroghazi JR, Ju KS, Yu X, Evans BS, Lee J, Metcalf WW.** 2014. Cyanohydrin phosphonate natural product from *Streptomyces regensis*. J Nat Prod **77:**243-249.
3. **Evans BS, Zhao C, Gao J, Evans CM, Ju KS, Doroghazi JR, van der Donk WA, Kelleher NL, Metcalf WW.** 2013. Discovery of the antibiotic phosacetamycin via a new mass spectrometry-based method for phosphonic acid detection. ACS Chem Biol **8:**908-913.
